# KK2DP7 Stimulates CD11b^+^ Cell Populations in the Spleen to Elicit Trained Immunity for Anti‐Tumor Therapy

**DOI:** 10.1002/advs.202500032

**Published:** 2025-03-27

**Authors:** Rui Zhang, Lin Tang, Yusi Wang, Xuejing Zhou, Zhenyu Ding, Li Yang

**Affiliations:** ^1^ Department of Biotherapy Cancer Center and State Key Laboratory of Biotherapy West China Hospital Sichuan University Chengdu 610041 China

**Keywords:** anti‐tumor therapy, CD11b+ cells, congenital immunity, training immunity, tumor immunotherapy

## Abstract

The induction of trained immunity for anti‐tumor therapy represents an emerging frontier in immunotherapy research, though its mechanistic underpinnings remain poorly understood. Adjuvant‐induced trained innate immune responses constitute a critical yet underexplored component of adjuvant mechanisms of action. Here, KK2DP7, a dendrimer‐structured peptide derived from the immunomodulatory antimicrobial peptide DP7 (VQWRIRVAVIRK) is employed, as a model adjuvant to establish standardized protocols for investigating adjuvant efficacy and mechanisms in enhancing anti‐tumor immunity via trained immunity. Initial studies revealed that KK2DP7 administration significantly delayed tumor growth post‐inoculation in murine models. The comprehensive analysis demonstrated that splenic cells exhibited cardinal features of trained immunity, whereas splenectomized mice exhibited complete loss of this protective effect. Strikingly, the adoptive transfer of CD11b^+^ cells isolated from the non‐lymphoid splenic compartment of KK2DP7‐trained mice to naïve recipients conferred robust tumor suppression. Mechanistic investigations linked this phenomenon to TLR2‐IRF7 axis activation and epigenetic reprogramming of CD11b^+^ cells, as evidenced by chromatin accessibility assays and histone modification profiling. These findings not only unveil a novel therapeutically actionable dimension of trained immunity, centered on spleen‐resident CD11b^+^ cell reprogramming but also establish a standardized protocol framework for systematically investigating adjuvant mechanisms in the context of trained innate immunity.

## Graphical abstract

This study introduces KK2DP7, a dendrimer‐structured peptide adjuvant, as a tool to decode trained immunity's role in antitumor therapy. Splenic CD11b+ cells mediate tumor suppression via TLR2‐IRF7 activation and epigenetic reprogramming, validated by adoptive transfer experiments. Splenectomy abolishes protection, while chromatin accessibility assays reveal mechanistic insights. This work establishes standardized protocols to dissect adjuvant‐driven trained immunity, advancing its therapeutic exploitation.

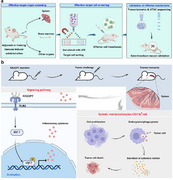

## 1 Introduction

In recent years, deepened understanding of immune system functions has highlighted a novel subset of immune responses termed “trained immunity”. This concept has garnered significant attention as a research hotspot due to its therapeutic potential in chronic diseases, including cancer.^[^ ^1^ ^]^ Trained immunity, also referred to as “innate immune memory” or “immune training”, describes the immune system's capacity to mount enhanced responses to subsequent challenges following initial exposure to microbial components or immune stimuli. Unlike traditional acquired immunity, trained immunity confers long‐term memory and sustains heightened responsiveness without requiring antigen‐specific re‐exposure.^[^ ^1^, ^2^ ^]^ Mechanistically, trained immunity involves immune cell priming through recognition of conserved molecular patterns (e.g., pathogen‐associated [PAMPs] or damage‐associated [DAMPs] molecular patterns), enabling rapid and potent responses to future stimuli.^[^ ^1^, ^2^ ^]^ This process not only enhances the speed and intensity of immune reaction but also expands pathogen recognition breadth, thereby amplifying overall immune system efficacy. Underlying these effects are metabolic adaptations, epigenetic remodeling, and transcriptional reprogramming.^[^ ^3^ ^]^ While trained immunity has been extensively studied in infectious and inflammatory diseases,^[^ ^1^, ^4^ ^]^ its translational potential in oncology is increasingly recognized. Recent preclinical studies suggest that trained immunity may be leveraged to suppress tumor progression,^[^ ^5^ ^]^ offering a promising frontier for cancer immunotherapy.

Numerous biological agents, particularly polysaccharide β‐glucans derived from fungi, have the ability to induce trained immunity. Extensive research has investigated this immunomodulatory potential in β‐glucans from *Candida albicans (C. albicans)* and *Trametes versicolor (T. versicolor)*, as well as those extracted from *Saccharomyces cerevisiae (S. cerevisiae)*.^[^ ^5^, ^6^ ^]^ While β‐glucan has established applications as a cancer immunomodulator, its therapeutic utilization through trained immunity mechanisms represents an emerging frontier in oncology research.^[^ ^1^, ^6^, ^7^ ^]^ The β‐glucan‐mediated cellular training effect initiates epigenetic reprogramming within bone marrow‐derived cells, specifically monocytes and macrophages. This reprogramming enhances their inflammatory responsiveness and antimicrobial capabilities.^[^ ^5^, ^7^ ^]^ Notably, trained immunity has demonstrated protective efficacy across species, conferring rabies resistance in canines and improving survival against lethal challenges of *(C. albicans)* and *Mycobacterium tuberculosis (M. tuberculosis)* in murine models.^[^ ^8^ ^]^ This nonspecific protective phenomenon, which extends to preventing secondary infections, positions trained immunity as a promising therapeutic paradigm for chronic disease management, including oncological applications. Despite growing academic recognition of trained immunity concepts, their mechanistic foundations remain incompletely characterized. Current understanding suggests the involvement of complex interplay between innate and adaptive immune cell populations, coupled with intercellular signaling pathways. This knowledge gap underscores the critical need for two parallel investigations: 1) development of safe immunomodulatory protocols for trained immunity induction, and 2) comprehensive elucidation of its molecular and cellular mechanisms. Adjuvant‐induced trained immune responses constitute a key component of adjuvant's multifaceted mechanisms of action. Recent studies have revealed that diverse adjuvants—including aluminum salts and Toll‐like receptor (TLR) agonists such as poly(I:C ) and CpG oligodeoxynucleotides—exert their effects, at least partially, through trained immunity.^[^ ^9^ ^]^ Notably, prior research identified KK2DP7, a dendrimer peptide derived from the immunomodulatory antimicrobial peptide DP7 (VQWRIRVAVIRK), as a TLR agonist with modestly enhanced adjuvant efficacy compared to conventional counterparts.^[^ ^10^ ^]^ Building on this evidence, we selected KK2DP7 as a model adjuvant to establish in vivo experimental protocols for systematically investigating how adjuvants leverage trained immunity to potentiate anti‐tumor immune responses, with a parallel emphasis on elucidating their mechanistic underpinnings.

Initial experiments demonstrated that the direct injection of KK2DP7 effectively retarded the growth of diverse mouse tumor types. Delving deeper, we observed that post‐injection with KK2DP7, mouse spleen cells exhibited a marked elevation in the secretion of cytokines associated with trained immunity upon secondary stimulation with LPS. Subsequently, we hypothesized that KK2DP7 might be training the splenic cells to mount potent antitumor immune responses. To substantiate this hypothesis, we surgically excised the spleen from mice and observed that the injection of KK2DP7 failed to attenuate tumor growth in these mice. This finding suggests that the antitumor effects of KK2DP7‐induced trained immunity are intricately linked to the cells residing in the mouse spleen. Furthermore, utilizing lymphocyte isolation techniques, flow cytometry, and magnetic bead sorting, we confirmed that KK2DP7 triggers the secretion of characteristic cytokines associated with trained immunity specifically from CD11b^+^ cells residing in the non‐lymphocyte fraction of the spleen. Furthermore, the transplantation of CD11b^+^ cells from the non‐lymphoid compartment of KK2DP7‐trained mice into naïve mice conferred antitumor growth capabilities in the recipient mice. This observation implies that KK2DP7 elicits antitumor potential in CD11b^+^ cells residing within the non‐lymphoid layer of the mouse spleen. To delve into the underlying mechanisms, we conducted transcriptome and ATAC sequencing, along with validation studies, on CD11b^+^ cells conditioned with KK2DP7. Our analysis revealed the activation of immune response and cytokine secretion pathways in these cells. Notably, CD11b^+^ cells exhibited marked proliferation, and their phagocytic and tumoricidal abilities were significantly augmented. Further exploration revealed that phenotypic alterations in CD11b^+^ cells are orchestrated by the TLR2‐IRF7 signaling axis. In mice lacking TLR2 and IRF7, the antitumor potency of KK2DP7‐induced trained immunity was attenuated. Intriguingly, the trained immunity conferred by KK2DP7 can be synergistically harnessed with immunotherapy utilizing immune checkpoint inhibitors to achieve superior antitumor outcomes. Our findings underscore a novel and therapeutically pertinent antitumor dimension of trained immunity, involving the strategic reprogramming of CD11b^+^ cells within the non‐lymphoid compartment of the spleen. Additionally, we provide a standard set of procedures for elucidating the mechanism of action of adjuvants or training immune agonists in inducing innate immune responses.

## 2 Results

### 2.1 KK2DP7 Helps Mice Resist the Growth of Multiple Tumors

**Figure 1:**
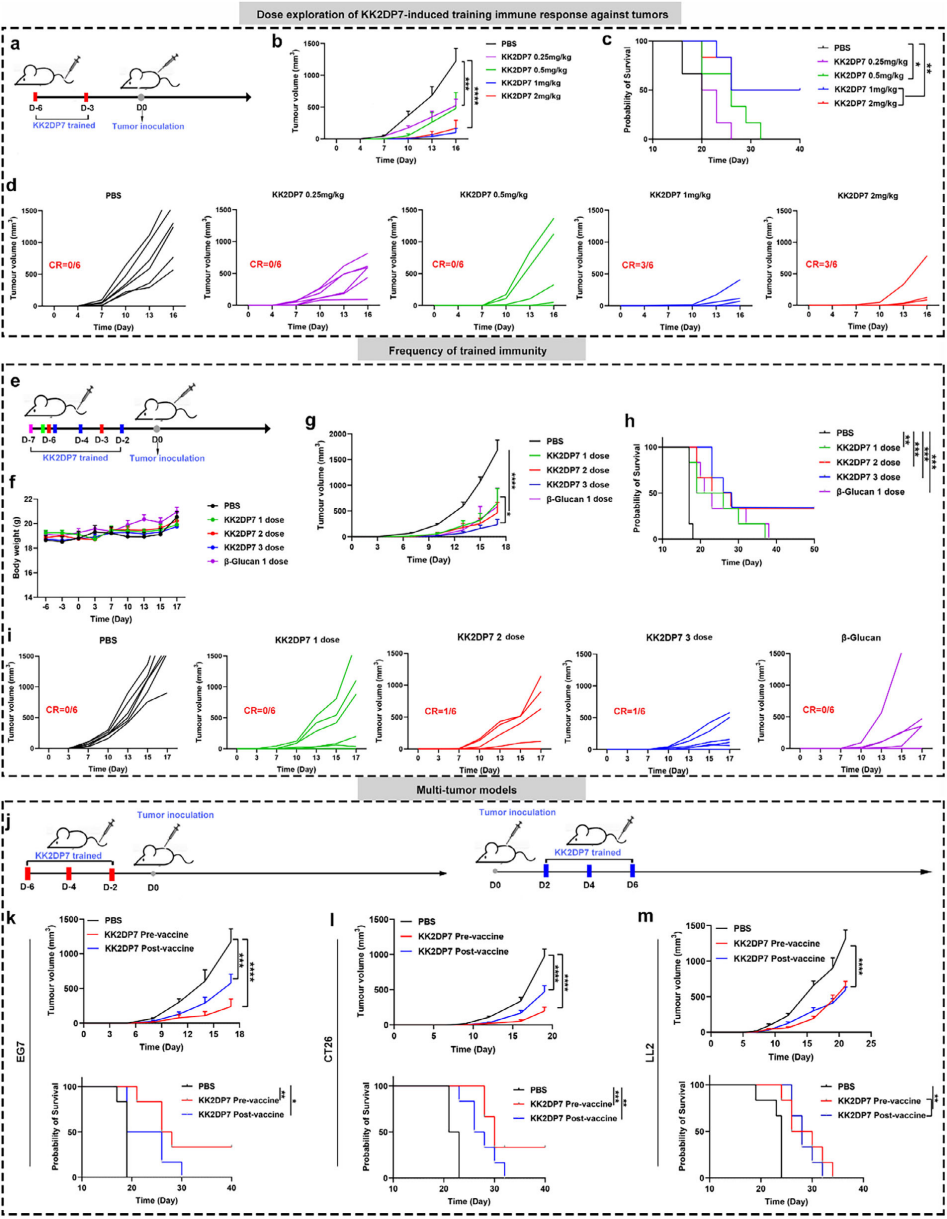
The administration of KK2DP7 has been shown to enhance the resistance of mice to multiple tumor growths. a) A schematic diagram outlining the experimental design for exploring the dose‐dependent induction of a training immune response against tumors is provided. b) The average tumor growth curve for mice administered different doses of KK2DP7 is shown (n = 6). c) Survival rates of mice in different groups after receiving various doses of KK2DP7 are presented (n = 6). d) Individual tumor growth curves corresponding to b are displayed. e) An experimental design diagram comparing the frequency of KK2DP7 administration in inducing training immunity and its anti‐tumor effects is shown. f) Body weights of mice in different groups with varying administration frequencies are reported (n = 6). g) The average tumor growth curve for mice treated with KK2DP7 at different frequencies is presented (n = 6). h) Survival rates of mice in different groups after KK2DP7 administration at various frequencies are shown (n = 6). i) Individual tumor growth curves corresponding to g are provided. j) A diagram of the experimental design for evaluating the anti‐tumor effects of pre‐ and post‐administration of KK2DP7 in multiple tumor models is given. k–m) The average tumor growth curves and mouse survival rates following pre‐ and post‐administration of KK2DP7 in the EG7, CT26, and LL2 tumor models are presented (n = 6). All values presented in this figure are expressed as the mean ± S.D., unless otherwise indicated in the figure captions. Statistical analysis was performed using one‐way ANOVA with Dunnett's multiple comparison test. CR was defined as complete tumor disappearance. Survival curves were obtained using the Kaplan‐Meier method and compared by the log‐rank test. ^*^ *P* < 0.05, ^**^ *P* < 0.01, ^***^ *P* < 0.001, ^****^ *P* < 0.0001.

Trained immunity is the capacity of innate immune cells to undergo long‐lasting functional reprogramming in response to certain exogenous or endogenous stimuli. This enables them to respond to subsequent homologous or even heterologous infections with a form of non‐specific immune memory, even after returning to their resting state.^[^ ^1^, ^2^, ^4^ ^]^ To ascertain whether KK2DP7, which exerts immune regulatory effects, can be considered an agonist of trained immunity, we sought to validate whether pre‐injection of KK2DP7 could inhibit tumor growth in mice. To this end, we intravenously injected mice with KK2DP7 at doses of 0.25, 0.5, 1, and 2 mg kg^−1^ before tumor inoculation. Subsequently, tumors were inoculated subcutaneously in the mice. It was observed that as the dose of KK2DP7 increased, the tumor growth in mice gradually slowed down, and the survival period was significantly prolonged (**Figure** **1**a–d). Additionally, there were no abnormalities in the body weight of the mice (Figure S1a, Supporting Information). Subsequently, to identify the optimal effect and frequency of KK2DP7 as a trained immunity agonist for antitumor prophylaxis, we inoculated tumors after pre‐injecting KK2DP7 once, twice, or three times (Figure 1e). To verify that three administrations of prophylactic dosing were most effective, we used the recognized trained immunity agonist β‐glucan as a control. The antitumor effects of KK2DP7 in terms of suppressing tumor growth and prolonging the survival of tumor‐bearing mice were comparable to those of β‐glucan (Figure 1f–i). This indicates that KK2DP7 may be a novel agonist of trained immunity. To further confirm the effects of KK2DP7, we verified its ability to significantly inhibit the growth of various mouse tumors and prolong the survival of tumor‐bearing mice. This was achieved by pre‐injecting KK2DP7 before tumor inoculation and injecting KK2DP7 after tumor inoculation in mouse lymphoma EG7, mouse colon cancer CT26, and mouse lung cancer LL2 (Figure 1j–m). Furthermore, there were no discernible abnormalities in the body weight of the mice (Figure S1b–g, Supporting Information). Consequently, we initially hypothesized that KK2DP7 may be analogous to β‐glucan in its capacity to act as an agonist of trained immunity.

### 2.2 KK2DP7 Induces Trained Immune Response in Mouse Spleen Cells to Resist Tumors

**Figure 2:**
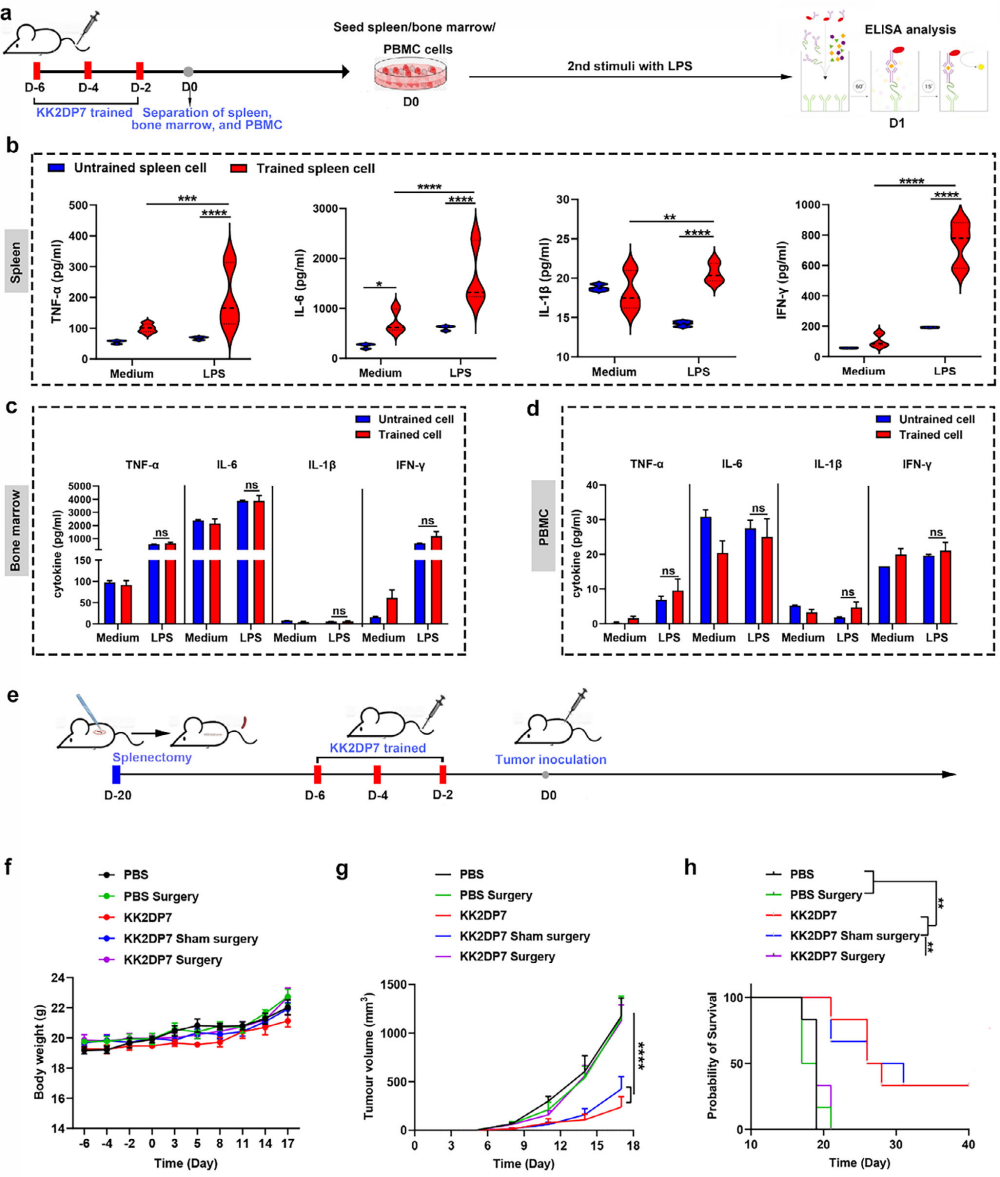
KK2DP7 induces cells in the spleen of mice to produce a training immune response leading to anti‐tumor resistance. a) Schematic diagram illustrating the verification of the site for the generation of the KK2DP7‐induced trained immune response. b) KK2DP7‐trained and LPS‐stimulated splenocytes exhibited significantly increased secretion of training‐immunity‐associated cytokines TNF‐α, IL‐1β, IL‐6, and IFN‐γ (n = 3). c,d) There were no significant changes in the secretion of training‐immunity‐associated cytokines (TNF‐α, IL‐1β, IL‐6, and IFN‐γ) in mouse bone marrow cells and PBMC after KK2DP7 training and LPS stimulation (n = 3). e) Experimental flowchart for verifying the anti‐tumor effect of KK2DP7 administration in a splenectomized mouse model. f) Body weight of mice in different groups were recorded (n = 6). g) The average tumor growth curve of mice in different groups is shown (n = 3). h) Survival rates of mice in different groups were assessed (n = 6). All values presented in this figure are expressed as the mean ± S.D., unless otherwise indicated in the figure captions. Statistical analysis was performed using one‐way ANOVA with Dunnett's multiple comparison test. Survival curves were obtained using the Kaplan–Meier method and compared by the log‐rank test. ^*^ *P* <  0.05, ^**^ *P* < 0.01, ^***^ *P* <  0.001, ^****^ *P* <  0.0001.

According to literature reports, the primary sites of trained immunity are the spleen and bone marrow.^[^ ^2^ ^]^ To ascertain whether the inhibitory effect of KK2DP7 on tumor growth is related to trained immunity, single‐cell suspensions were isolated from the spleen, bone marrow, and peripheral blood mononuclear cells (PBMCs) of mice following pre‐injection with KK2DP7. LPS was then added for secondary stimulation (**Figure** **2**a). The culture supernatants were collected, and secretion levels of characteristic cytokines of trained immunity, TNF‐α, IL‐6, IL‐1β, and IFN‐γ, were quantified. Results demonstrated that KK2DP7‐trained spleen cells exhibited markedly elevated cytokine secretion compared to control groups (untrained, LPS‐only stimulated, and trained but unstimulated) (Figure 2b). Comparison with the untreated tumor‐bearing group revealed no impact of spleen resection on tumor growth or survival time. However, in mice with spleen resection, KK2DP7‐trained immunity failed to inhibit tumor growth, as their tumor progression mirrored the PBS control group and was significantly faster than non‐resected mice (Figure 2f–h). To control for surgical effects, a sham surgery group (surgery without resection) was included. KK2DP7‐trained sham mice showed slower tumor growth and longer survival than the true surgery group (Figure 2f–h; Figure S1c, Supporting Information). These findings indicate that KK2DP7 acts as a trained immunity agonist, exerting antitumor effects via splenic cell training.

### 2.3 KK2DP7 Generates Anti‐Tumor Immunity by Inducing CD11b^+^ Cells in the Non‐Lymphocytic Layer of Mouse Spleen

**Figure 3:**
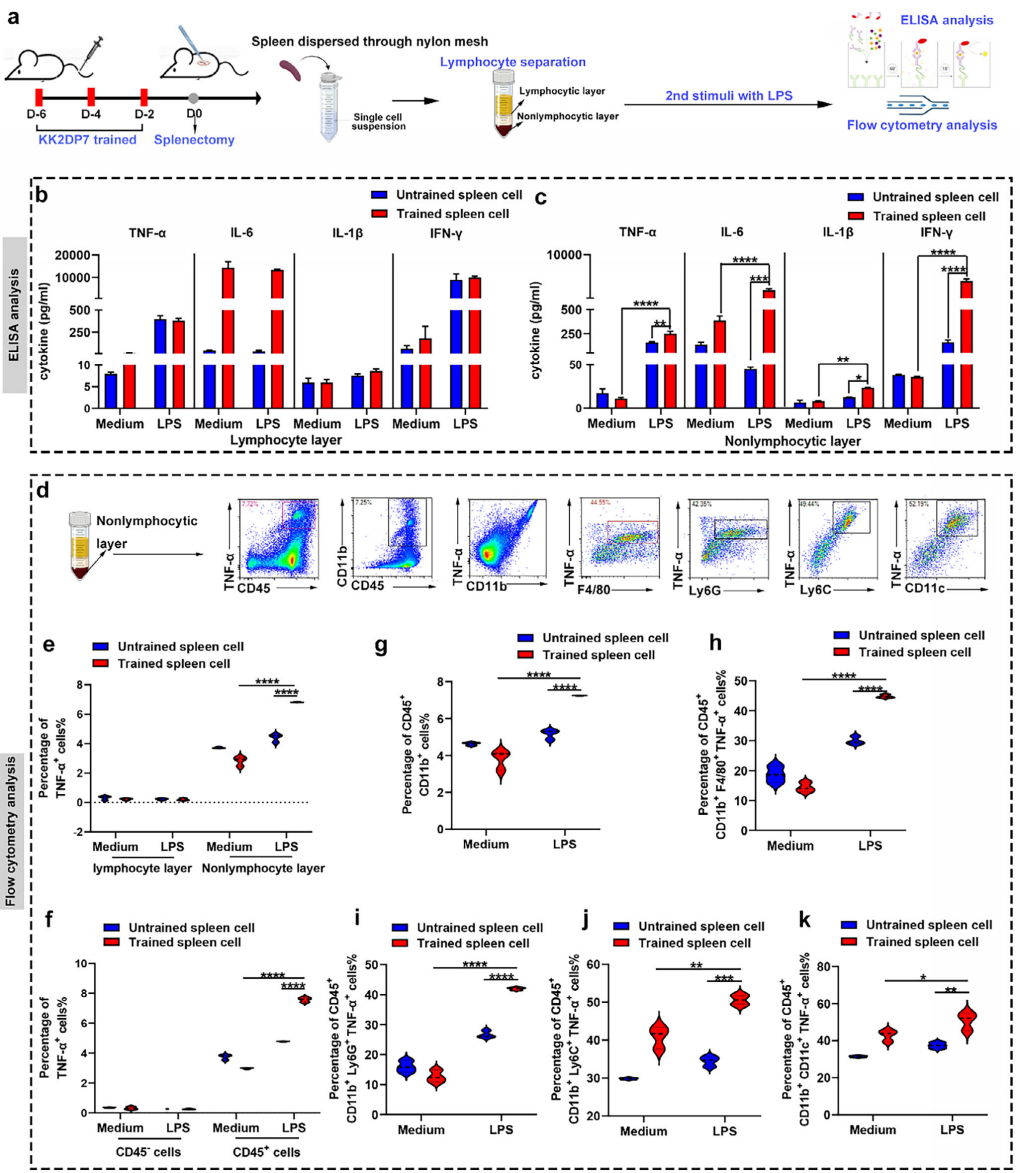
The target cells for training immunity induced by KK2DP7 are cells in the non‐lymphocyte layer of the spleen. a) Validation flowchart for KK2DP7‐induced training immunity in spleen target cell population. b) Cells in the splenic lymphocyte layer of mice trained with KK2DP7 and stimulated with LPS show no significant changes in the secretion of training‐immunity‐associated TNF‐α, IL‐1β, IL‐6, and IFN‐γ (n = 3). c) Cells in the splenic non‐lymphocyte layer of mice trained with KK2DP7 and stimulated with LPS exhibited a significant upregulation in the secretion of training‐immunity‐associated TNF‐α, IL‐1β, IL‐6, and IFN‐γ (n = 3). d) Representative flow cytometry plots of target cells in the non‐lymphocytic layer of mouse spleen are presented. e) TNF‐α production by cells in the non‐lymphocytic layer of the mouse spleen was significantly elevated after training with KK2DP7 and stimulation with LPS, whereas no changes in TNF‐α production were observed in cells in the lymphocytic layer (n = 3). f) TNF‐α production by CD45^+^ cells in the non‐lymphocytic layer of the mouse spleen was significantly elevated after training with KK2DP7 and stimulation with LPS, whereas no changes in TNF‐α production were observed in CD45^‐^ cells (n = 3). g) Following training with KK2DP7 and LPS stimulation, the proportion of CD45^+^CD11b^+^ cells in the non‐lymphocyte layer of the mouse spleen was found to be significantly upregulated (n = 3). h–k) Training with KK2DP7 and LPS stimulation led to a significant increase in the proportion of macrophages (CD11b^+^F4/80^+^), neutrophils (CD11b^+^Ly6G^+^), monocytes (CD11b^+^Ly6C^+^), and dendritic cells (CD11b^+^ CD11c^+^) expressing TNF‐α in the non‐lymphocytic layer of the mouse spleen (n = 3). All values presented in this figure are expressed as the mean ± S.D., unless otherwise indicated in the figure captions. Statistical analysis was performed using one‐way ANOVA with Dunnett's multiple comparison test. ^*^ *P* < 0.05, ^**^ *P* < 0.01, ^***^ *P* < 0.001, ^****^ *P* < 0.0001.

To ascertain which cell types within the spleen are responsive to KK2DP7, the spleen was first separated into lymphocyte and non‐lymphocyte layers using a lymphocyte separation medium. Subsequently, LPS stimulation was applied, and cytokines in the supernatant of the culture medium were detected using ELISA (**Figure** **3**a). The results demonstrated that there was no discernible difference in the secretion levels of four cytokines between the lymphocyte layer cells that had been trained with KK2DP7 and stimulated with LPS and the control group. However, the non‐lymphocyte layer cells that had been trained with KK2DP7 and stimulated with LPS exhibited a significant upregulation compared to the group that had not been trained but had been stimulated with LPS and the group that had been trained but not stimulated with LPS (Figure 3b,c). Furthermore, when spleen lymphocytes and non‐lymphocytes were isolated from normal mice in vitro, trained three times with KK2DP7, and then stimulated with LPS, similar results were observed to those observed in vivo training, indicating that the non‐lymphocyte layer cells in the spleen exhibited characteristic upregulation of cytokine secretion after trained immunity (Figure S2a–c, Supporting Information). These findings indicate that the target cells trained by KK2DP7 are located in the non‐lymphocyte layer of the spleen.

In light of these findings, flow cytometry experiments were conducted to assess the expression levels of TNF‐α in the lymphocyte and non‐lymphocyte layers of the spleen cells from mice trained with KK2DP7 and stimulated with LPS (Figure 3d; Figure S3a,b, Supporting Information). The results demonstrated that there was no significant TNF‐α expression observed in the spleen lymphocyte layer cells. However, the expression of TNF‐α in the non‐lymphocyte layer cells of the spleen exhibited characteristic features of trained immunity, with significantly higher expression levels after training and LPS stimulation compared to the groups that were either not trained but stimulated with LPS or trained but not subjected to secondary stimulation (Figure 3e). Subsequently, the non‐lymphocyte layer cells of the spleen were further categorized into immune and non‐immune cells based on CD45 expression. Notably, no significant TNF‐α expression was observed in the CD45^−^ cell population. In contrast, the expression of TNF‐α in the CD45^+^ cell population demonstrated characteristic features of trained immunity (Figure 3f; Figure S3c, Supporting Information). Furthermore, the CD45^+^ cells were divided into CD11b^+^ and CD11b^−^ cell populations. It is noteworthy that the CD11b^+^ cell population exhibited the most prominent TNF‐α expression, which is characteristic of trained immunity (Figure 3d; Figure S3b,c, Supporting Information). Furthermore, the proportion of CD11b^+^ cells was significantly elevated following training and LPS stimulation (Figure 3g; Figure S3c, Supporting Information). To gain further insight, we proceeded to analyze the various cell types within the CD11b^+^ population, including macrophages, monocytes, neutrophils, and dendritic cells. The findings revealed that the expression of TNF‐α in all these cell types exhibited characteristic features of trained immunity (Figure 3h–k; Figure S3b,c, Supporting Information). These findings indicate that the target cells of KK2DP7‐induced trained immunity are the CD11b^+^ cell population within the non‐lymphocyte layer of the spleen.

**Figure 4:**
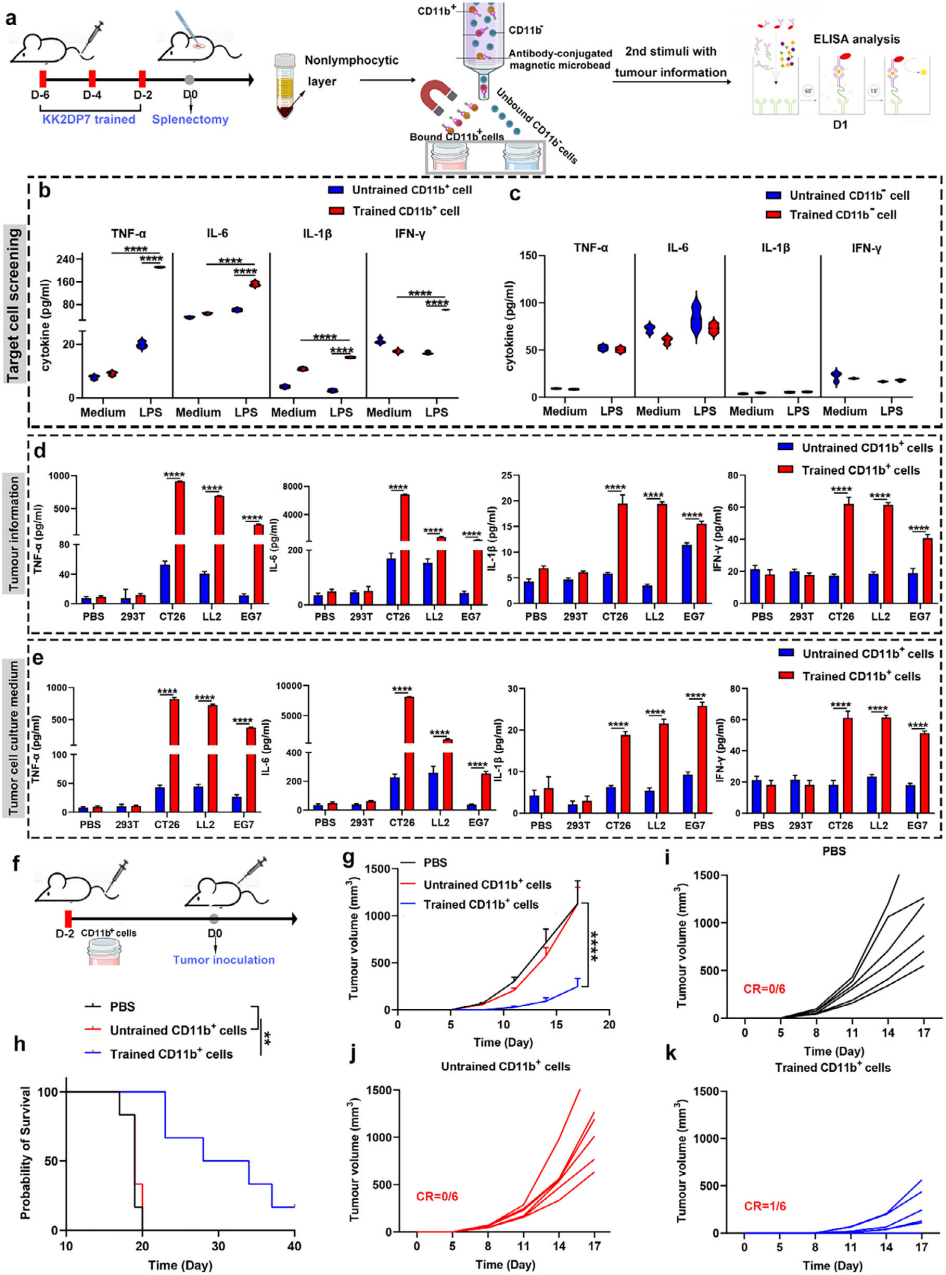
KK2DP7 induces the training of immunotherapy against tumors by activating CD11b^+^ cells in the non‐lymphocytic layer of the spleen. a) The validation flowchart for KK2DP7‐induced training immunity in the spleen target cell population is presented below. b) CD11b^+^ cells in the splenic non‐lymphocytic layer of mice trained with KK2DP7 and stimulated with LPS exhibited a significant upregulation of TNF‐α, IL‐1β, IL‐6, and IFN‐γ secretion, which is indicative of training immunity (n = 3). c) CD11b^‐^ cells in the splenic nonlymphocytic layer of mice trained with KK2DP7 and stimulated with LPS did not exhibit any significant changes in the secretion of training‐immunity‐associated TNF‐α, IL‐1β, IL‐6, and IFN‐γ (n = 3). d,e) The CD11b^+^ cells in the non‐lymphocyte layer of the spleen, which were trained with KK2DP7 and stimulated by tumor cells or tumor cell culture supernatant, demonstrated a significant upregulation of immune‐related TNF‐α, IL‐1β, IL‐6, and IFN‐γ secretion (n = 3). f) A flowchart of the adoptive transfer of CD11b^+^ cells from the non‐lymphocyte layer of the spleen, following training with KK2DP7, for antitumor application is provided. g) The average tumor growth curve of mice in different groups is shown (n = 6). h) The survival rates of mice in different groups are presented (n = 6). i–k) Individual tumor growth curves from g are displayed. All values presented in this figure are expressed as the mean ± S.D., unless otherwise indicated in the figure captions. Statistical analysis was performed using one‐way ANOVA with Dunnett's multiple comparison test. Survival curves were obtained using the Kaplan–Meier method and compared by the log‐rank test. ^****^ *P* < 0.0001.

To further validate the aforementioned results, we employed magnetic bead sorting technology to separate the non‐lymphocyte cells from the spleen of mice trained with KK2DP7 into CD11b^−^ and CD11b^+^ subpopulations. Similarly, non‐lymphocyte cells from the spleen of normal mice were also sorted into CD11b^−^ and CD11b^+^ subpopulations for in vitro experiments. LPS stimulation was applied to both groups, with or without prior KK2DP7 training, to assess the expression of training‐associated immune cytokines in the culture supernatant (**Figure** **4**a; Figure S4a, Supporting Information). The results consistently demonstrated that, following KK2DP7 training, the CD11b^−^ subpopulation failed to exhibit the characteristic immune training responses observed in the CD11b^+^ subpopulation upon stimulation. The CD11b^+^ subpopulation displayed significant elevations in the expression of TNF‐α, IL‐6, IL‐1β, and IFN‐γ following LPS stimulation (Figure 4b,c; Figure S4b,c, Supporting Information). This observation further corroborates the hypothesis that the target cells of KK2DP7‐induced immune training are the CD11b^+^ subpopulation within the non‐lymphocyte cells of the spleen. Subsequently, we replaced LPS with normal cells, tumor cells, and culture supernatants derived from both normal and tumor cells to stimulate the CD11b^+^ subpopulation from the non‐lymphocyte layer of the spleen following KK2DP7 training. The results demonstrated that CD11b^+^ cells did not elicit the characteristic immune training responses upon stimulation with normal cells or their culture supernatants. However, stimulation with various tumor cells or their culture supernatants triggered the secretion of cytokines characteristic of immune training (Figure 4d,e). These findings indicate that the immune training induced by KK2DP7 is capable of eliciting characteristic cytokine secretion in response to a range of danger signals while remaining unresponsive to normal cells.

The aforementioned validations have led to the identification of the CD11b^+^ subpopulation within the non‐lymphocyte layer of the spleen as the target cells of KK2DP7‐induced immune training. To provide more conclusive evidence, the KK2DP7‐trained CD11b^+^ subpopulation was adoptively transferred into naive mice. The transfer conferred tumor resistance in the untreated mice upon tumor challenge, manifesting as slower tumor growth and significantly prolonged survival (Figure 4f–k). These findings provide stronger evidence to support the hypothesis that CD11b^+^ cells within the non‐lymphocyte layer of the spleen are the target cells of KK2DP7‐induced immune training.

### 2.4 Upon Exposure to KK2DP7, CD11b^+^ Cells Exhibited Profound Alterations at Both the Transcriptomic and ATAC‐seq Levels

**Figure 5:**
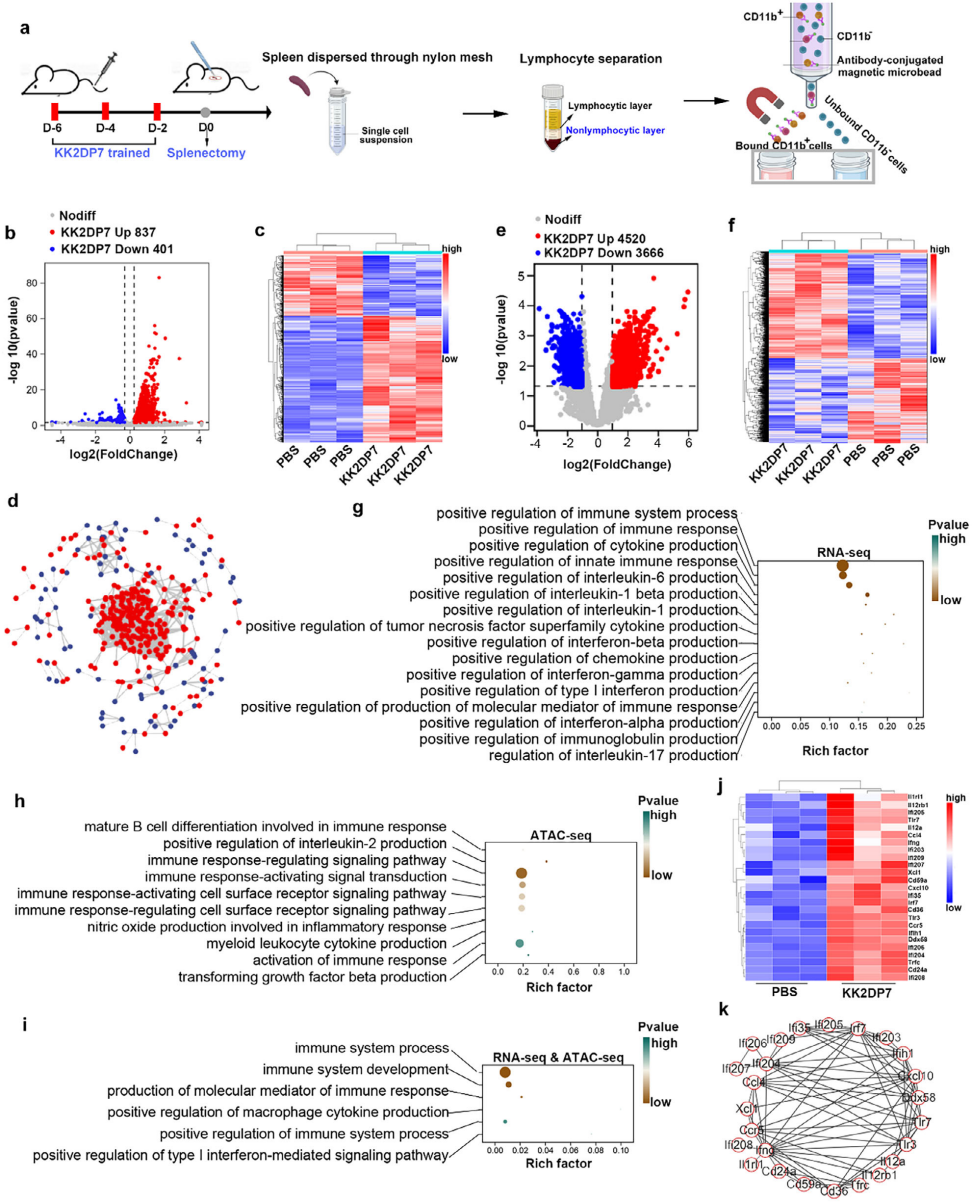
Significant differences were observed in the transcriptome and ATAC sequencing of CD11b^+^ cells in the non‐lymphocyte layer of the mouse spleen following KK2DP7 treatment. a) Flowchart illustrating the target cell acquisition process for sequencing. b) Differential gene expression in CD11b^+^ cells pretreated with KK2DP7 compared with those from PBS‐treated mice in RNA‐sequence (n=3). A volcano plot is presented, displaying the distribution of the adjusted p values (log10(padj)) and fold changes (log2 fold change). c) A heatmap of genes from KK2DP7‐treated mice as compared with PBS‐treated mice in RNA‐sequence is also included. d) An interaction network diagram of all changing genes in b is provided, as well as e) a differential peak expression in mice pre‐treated with KK2DP7 compared with from PBS‐treated mice in ATAC‐sequence. A volcano plot is presented, which shows the distribution of the adjusted p values (log10(padj)) and fold changes (log2 fold change). f) A heatmap is also provided, which shows the genes from KK2DP7‐treated mice as compared with PBS‐treated mice in ATAC sequence analysis. g) Gene ontology (GO) enrichment of immune‐related pathways in KK2DP7‐treated mice compared to PBS‐treated mice in assay for RNA‐seq analysis. h) Gene ontology (GO) enrichment of immune‐related pathways in KK2DP7‐treated mice compared to PBS‐treated mice in assay for transposase‐accessible chromatin sequencing (ATAC‐seq). i) Among the genes with shared changes in both RNA‐seq and ATAC‐seq, GO enrichment of immune‐related pathways in KK2DP7‐treated mice compared to PBS‐treated mice is analyzed. j) An expression heatmap of immune‐related genes that are commonly varied in both RNA‐seq and ATAC‐seq is provided. k) An interaction network diagram of all genes shown in j is included.

The existing literature indicates that the mechanisms underlying trained immunity are associated with changes in metabolism, epigenetics, and transcriptomic profiles.^[^ ^3^, ^5^, ^11^ ^]^ A number of studies have employed a combined approach of transcriptome and ATAC sequencing to elucidate the mechanisms of trained immunity induced by other agonists. To further validate the mechanism of KK2DP7‐induced trained immunity in the CD11b‐positive subpopulation of the non‐lymphocyte layer of the spleen against secondary infection, we conducted transcriptome and ATAC sequencing of CD11b^+^ cells from this layer before and after KK2DP7 training (**Figure** **5**a). The results demonstrated significant differences at both the RNA and ATAC levels following KK2DP7 training (Figure 5b–f). In particular, 837 genes were found to be significantly upregulated, while 421 genes were downregulated in the RNA sequencing data. Based on interaction patterns and fold changes, the upregulated genes appeared to occupy a central position within the transcriptome (Figure 5b,c). In the ATAC sequencing data, 4520 peaks were found to be significantly upregulated, while 3666 peaks were downregulated (Figure 5e,f). Gene Ontology (GO) functional enrichment analysis of the differentially expressed genes revealed that functions related to positive regulation of immune responses, innate immune responses, and cytokine production were enriched in the RNA sequencing data (Figure 5g). In the ATAC sequencing data, functions related to immune response activation and cytokine production were significantly enriched (Figure 5h). Upon intersecting the observed changes in both sequencing modalities, GO functional enrichment revealed the enrichment of functions related to positive regulation of immune responses, cytokine production, and type I interferon regulation (Figure 5i). Among these, several genes, such as IRF7 and the Ifi gene family, were significantly upregulated and displayed clear interaction patterns (Figure 5j,k). These initial sequencing results suggest that KK2DP7 training positively regulates the immune system.

### 2.5 Proliferation, Phagocytosis, and Toxicity of CD11b^+^ Cells in the Non‐Lymphocytic Layer of the Spleen were Activated after KK2DP7 Training

**Figure 6:**
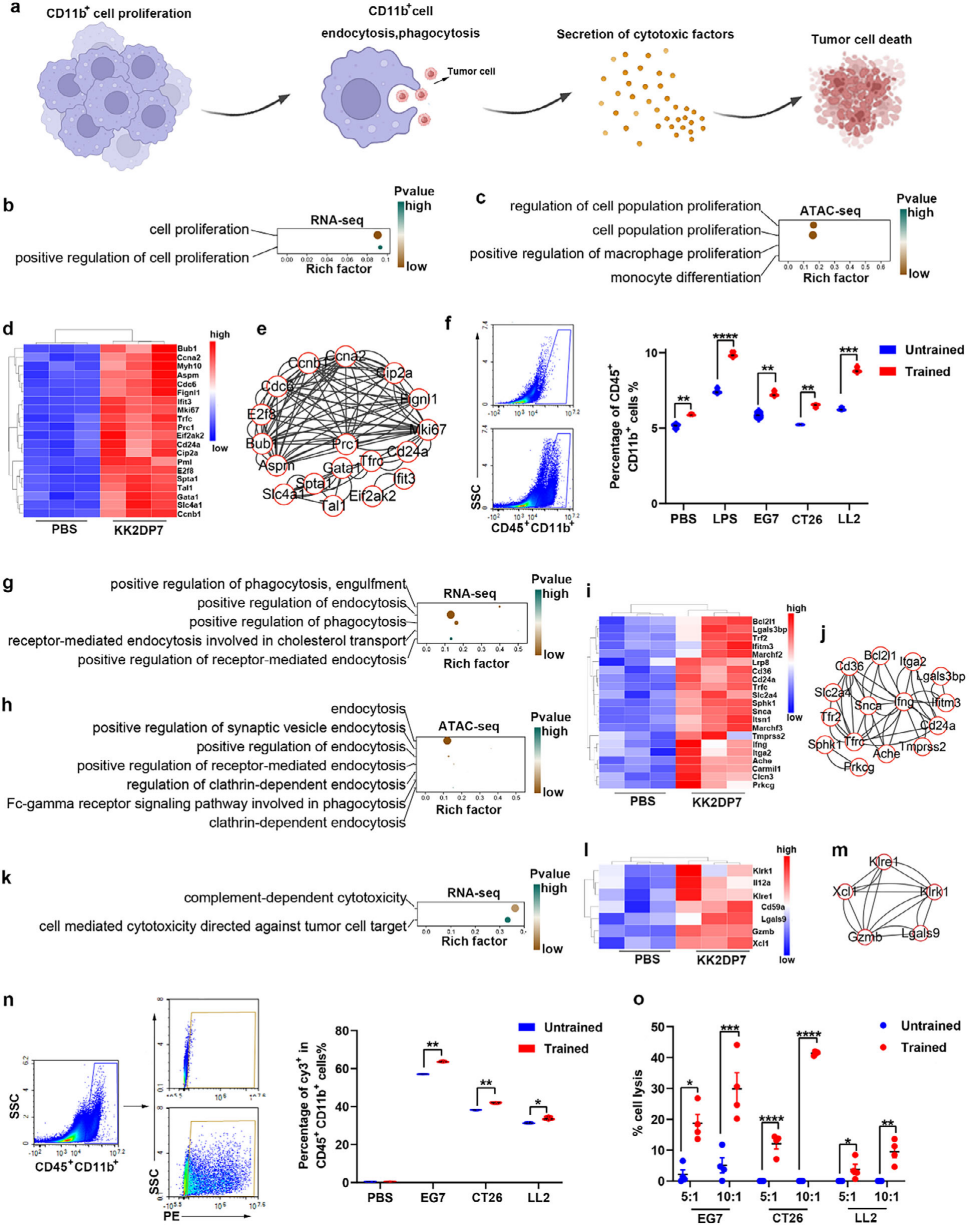
The proliferation, phagocytosis and cytotoxicity of CD11b^+^ cells in the non‐lymphocytic layer of the spleen are significantly enhanced following KK2DP7 training. a) Schematic diagram illustrating validated cell functions. b‐c) Gene Ontology (GO) enrichment analysis of cell proliferation‐related pathways in mice treated with KK2DP7 revealed a significant upregulation compared to mice treated with PBS, as evidenced by RNA and ATAC sequencing. d‐e) The expression heatmap of cell proliferation‐related genes and the interaction network diagram of all genes in the heatmap demonstrated a clear distinction between the two groups. f) The results of flow cytometry demonstrated a significant increase in the proliferation of CD11b^+^ cells in the non‐lymphocyte layer of the spleen following KK2DP7 training and secondary stimulation. g‐h) GO enrichment of cell endocytosis‐related pathways in KK2DP7‐treated mice compared to PBS‐treated mice in RNA and ATAC sequencing. i) Expression heatmap of cell endocytosis‐related genes. j) Network diagram of all genes in i. k) GO enrichment of cell cytotoxicity‐related pathways in KK2DP7‐treated mice compared to PBS‐treated mice in RNA sequencing. l) Expression heatmap of cell cytotoxicity‐related genes. m) Network diagram of all genes in l. n) The results of flow cytometry demonstrated that following KK2DP7 training, the capacity of CD11b^+^ cells in the non‐lymphocyte layer of the spleen to phagocytose tumor cells was significantly enhanced (n=3). o) The results of flow cytometry indicated that the capacity of CD11b^+^ cells in the non‐lymphocyte layer of the spleen to kill tumors was significantly enhanced following KK2DP7 training (n=3). All values presented in this figure are expressed as the mean ± s.d., unless otherwise indicated in the figure captions. Statistical analysis was performed using one‐way ANOVA with Dunnett's multiple comparison test. In contrast, a student's t‐test was utilized for two‐group comparisons. *P < 0.05, **P < 0.01, ***P < 0.001, ****P < 0.0001.

Further analysis of the additional functions of the differentially expressed genes revealed significant enrichment of three functions: cell proliferation, phagocytosis, and cytotoxicity. With regard to cell proliferation, both the RNA and ATAC data indicated an enrichment of functions that positively regulate cell proliferation (**Figure** **6**b,c). A number of related genes, including the representative gene Mki67, were found to be significantly upregulated and exhibited clear interactions among them (Figure 6d,e). Flow cytometry analysis of cell proliferation before and after training, as well as following different secondary stimuli, revealed that both KK2DP7 training and subsequent secondary stimulation with LPS or tumor cells induced proliferation in the CD11b^+^ population of the non‐lymphocyte layer of the spleen (Figure 6f). In terms of phagocytosis, both RNA and ATAC data demonstrated an enrichment of functions positively regulating endocytosis and phagocytosis (Figure 6g,h). A number of related genes, including CD36 and Itga2, exhibited significant upregulation and displayed clear gene–gene interactions (Figure 6i,j). Co‐incubation experiments of spleen non‐lymphocyte layer cells before and after training with fluorescently labeled tumor cells, followed by flow cytometry analysis of CD11b^+^ cell phagocytosis efficiency, revealed that the ability of CD11b^+^ cells to phagocytose tumor cells was significantly higher after KK2DP7 training compared to the untrained group (Figure 6n). Furthermore, RNA data indicated enrichment of functions related to cytotoxicity (Figure 6k). A number of related genes, including Gzmb, exhibited significant upregulation and displayed clear gene–gene interactions (Figure 6l,m). Co‐incubation experiments of CD11b^+^ cells from the non‐lymphocyte layer of the spleen before and after KK2DP7 training with tumor cells at ratios of 5:1 and 10:1, followed by LDH release detection using an LDH kit, demonstrated that the cytotoxic ability of CD11b^+^ cells against tumor cells was significantly higher after KK2DP7 training compared to the untrained group (Figure 6o). In conclusion, these results demonstrate that the functions of cell proliferation, phagocytosis, and cytotoxicity in CD11b^+^ cells from the non‐lymphocyte layer of the spleen in mice were significantly activated following KK2DP7 training (Figure 6a).

### 2.6 Antitumor Immune Reprogramming of CD11b^+^ Cells after KK2DP7 Training is Regulated by the TLR2‐IRF7 Pathway

**Figure 7:**
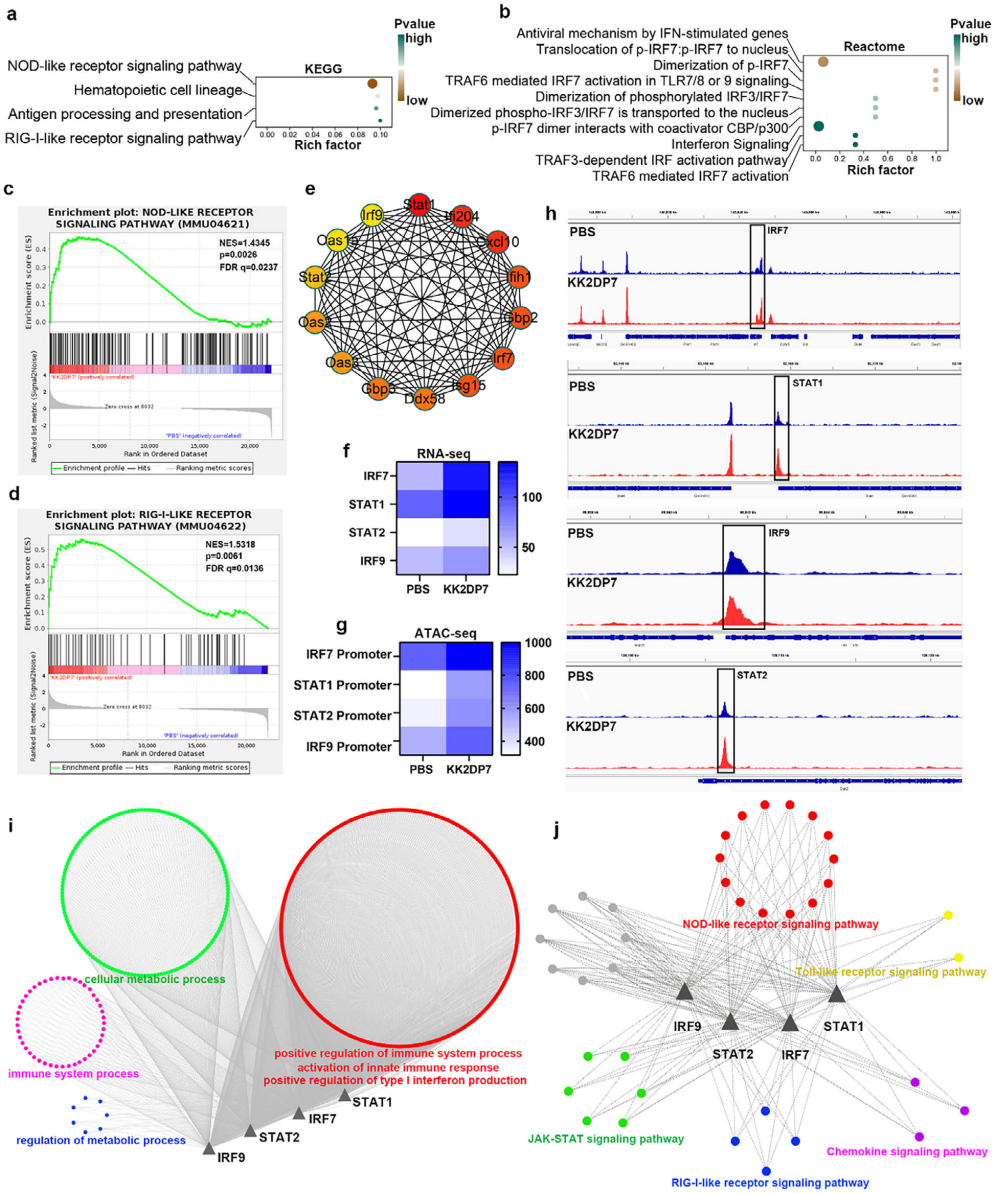
Transcriptome changes and epigenetic reprogramming induced by immune training in KK2DP7. a) Pathways of KEGG enrichment in CD11b^+^ cells of the non‐lymphocytic layer of the spleen in mice treated with KK2DP7. b) The interferon‐related pathway of Reactome enrichment in CD11b^+^ cells of the non‐lymphocytic layer of the spleen in mice treated with KK2DP7. c‐d) Gene set enrichment analysis (GSEA) was conducted on genes related to the NOD‐ and RIG‐I‐like receptor signal pathway in CD11b^+^ cells treated with KK2DP7 compared to those treated with PBS. The top core genes and their interactions in the NOD, RIG‐I, and interferon signaling pathways were displayed. Furthermore, a heatmap of transcription factor expression in RNA and ATAC sequences was generated. h) A genome browser track demonstrating differentially accessible regions associated with differential transcription factors. i) An analysis of GO functional networks regulated by differential transcription factors. j) An analysis of KEGG signaling pathway networks regulated by differential transcription factors.

In terms of signaling pathways, the KEGG enrichment results indicated that the NOD‐like receptor signaling pathway and the RIG‐I‐like receptor signaling pathway were activated in target cells following KK2DP7 training (**Figure** **7**a). Reactome enrichment analysis demonstrated the activation of interferon‐related pathways (Figure 7b). The GSEA analysis of the NOD‐like receptor signaling pathway and the RIG‐I‐like receptor signaling pathway confirmed significant activation of both pathways (Figure 7c,d). Cytoscape was employed for further visualization, with the top genes and their interaction networks within these signaling pathways exhibited. This revealed the presence of four crucial transcription factors: The following transcription factors were identified: Stat1, IRF7, STAT2, and IRF9 (Figure 7e). The expression levels of the four key transcription factors were found to be significantly upregulated in both the RNA and ATAC data sets (Figure 7f,g). Furthermore, the ATAC sequencing visualization results demonstrated observable differences in these transcription factors (Figure 7h). Transcriptional factor regulatory network analysis of the GO functions and KEGG pathways regulated by these four transcription factors revealed enrichment of functions such as positive regulation of immune response, positive regulation of innate immune response, and positive regulation of type I interferon activity in GO functions (Figure 7i). Furthermore, the NOD‐like receptor signaling pathway, RIG‐I‐like receptor signaling pathway, and Toll‐like receptor signaling pathway were identified as enriched in KEGG signaling pathways (Figure 7j). These findings corroborate the previously identified differences, thereby underscoring the pivotal role of these transcription factors.

**Figure 8:**
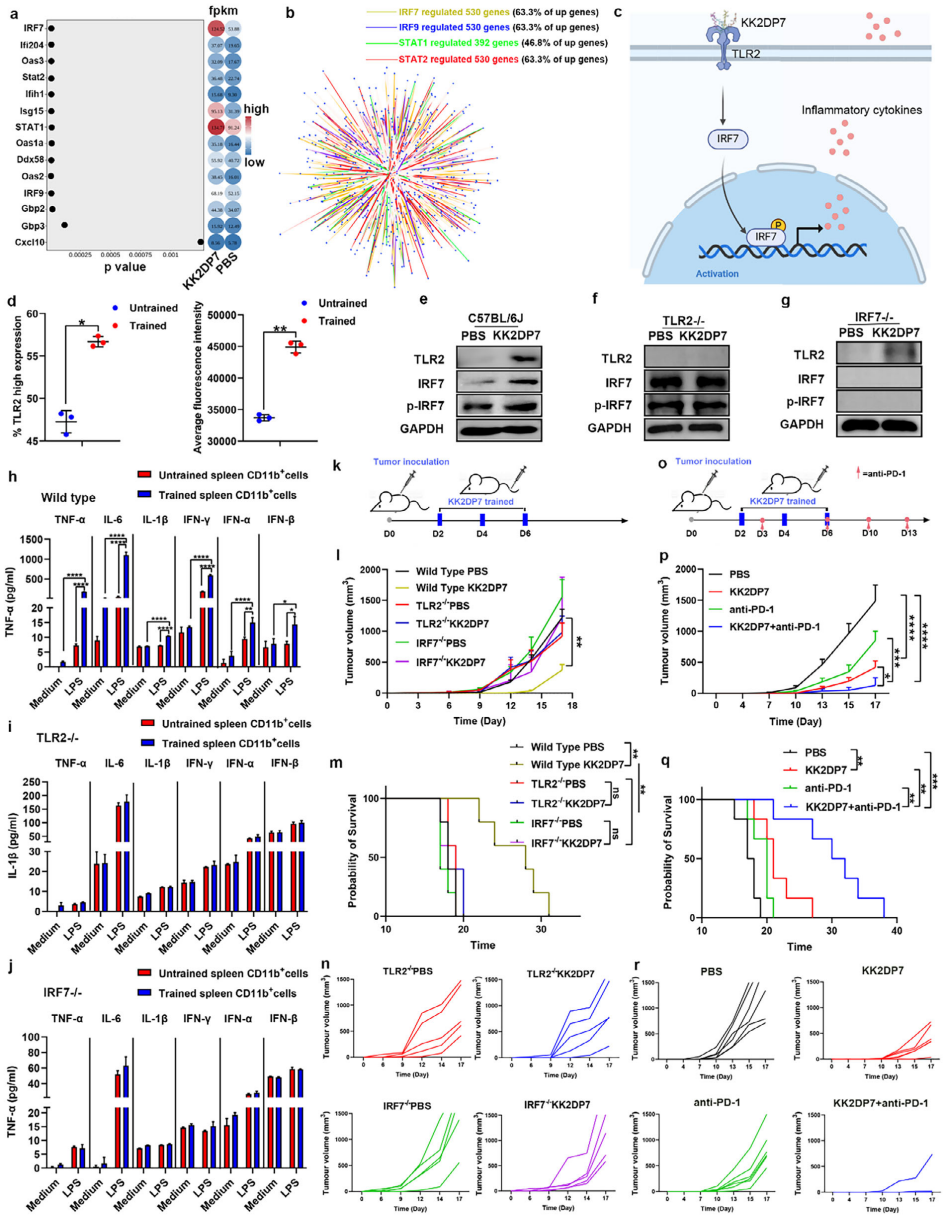
The induction of training immunity by KK2DP7 is dependent on the involvement of TLR2‐IRF7 signaling. a) Display of p‐values and expression levels of the top core genes in differential signaling pathways. b) Transcription factor regulatory gene network analysis diagram. c) Mechanistic map of KK2DP7's regulation of trained immunity. d) Up‐regulation of TLR2 expression levels in CD45^+^ CD11b^+^ cells in the non‐lymphocyte layer of mouse spleen after KK2DP7 training (n=3). e‐g) The validation of TLR2, IRF7, and p‐IRF7 expression before and after immune training with KK2DP7 in wild‐type mice, TLR2 knockout mice, and IRF7 knockout mice (n=3). h‐j) The CD11b^+^ cells in the splenic nonlymphocytic layer of TLR2 knockout mice and IRF7 knockout mice did not exhibit notable alterations in the secretion of immune‐associated cytokines following KK2DP7 induction. This was in stark contrast to the observed changes in wild‐type mice (n=3). k) Flowchart of inoculation and KK2DP7 administration in knockout mice. l) The average tumor growth curve of mice in different groups (n=6). m) The survival rates of mice in different groups. n) Individual tumor growth curves from l. o) Flow chart of KK2DP7 combined with anti‐PD‐1 antibody in the treatment of mouse tumors. p) The average tumor growth curve of mice in different groups (n=6). q) The survival rates of mice in different groups. r) Individual tumor growth curves from p. All values presented in this figure are expressed as the mean ± s.d., unless otherwise indicated in the figure captions. Statistical analysis was performed using one‐way ANOVA with Dunnett's multiple comparison test. In contrast, a student's t‐test was utilized for two‐group comparisons. Survival curves were obtained using the Kaplan–Meier method and compared by the log‐rank test. *P < 0.05, **P < 0.01, ***P < 0.001, ****P < 0.0001.

To further investigate the transcriptional changes, we performed a heatmap analysis of the fold change and significance p‐values for the selected transcription factors. IRF7 emerged as the factor with the highest fold change and the most significant difference (**Figure** **8**a). In addition, there exists a mutual regulatory relationship among these four transcription factors, with IRF7 serving as an upstream regulator of the other three.^[^ ^12^ ^]^ And IRF7 controls 63.3% of the genes altered in the RNA‐seq data (Figure 8b). Consequently, IRF7 was identified as a critical transcription factor for validation, and it was crucial to identify the upstream pathways regulating IRF7. Previously, several signaling pathways that interact with IRF7 were identified, including the NOD‐like receptor signaling pathway, RIG‐I‐like receptor signaling pathway, Toll‐like receptor signaling pathway, JAK‐STAT signaling pathway, and Chemokine signaling pathway (Figure 7g). Notably, the JAK‐STAT and Chemokine signaling pathways require activation by cytokines, suggesting that cytokines produced after IRF7 activation may further activate these pathways.^[^ ^13^ ^]^ In the case of the RIG‐I pathway, which requires activation by double‐stranded DNA and RNA,^[^ ^14^ ^]^ the absence of such structures in KK2DP7 suggests that cytokines generated following IRF7 activation might activate downstream components of the RIG‐I pathway related to interferon activation. Within the NOD‐like signaling pathway, while classical NOD1 and NOD2 did not exhibit significant changes, the activation was concentrated downstream of interferon secretion. With regard to the Toll‐like receptor signaling pathway, previous studies have demonstrated that KK2DP7 can activate TLR2 in dendritic cells (DCs) and promote their maturation through downstream signaling.^[^ ^10^, ^15^ ^]^ Since IRF7 is also a downstream gene of TLR2,^[^ ^16^ ^]^ it can be hypothesized that KK2DP7 might activate TLR2, leading to IRF7 activation and subsequent secretion of cytokines like interferons, which further activate other pathways to induce a trained antitumor immune response (Figure 8c).

To validate this hypothesis, we first analyzed the expression of TLR2 in splenic CD11b^+^ cells from non‐lymphoid layers before and after KK2DP7 training using flow cytometry. The results demonstrated a significant upregulation of TLR2 expression in the trained group in comparison to the control (Figure 8d). Subsequently, western blot and ELISA analyses were conducted on splenic CD11b^+^ cells from non‐lymphoid layers of normal mice, TLR2^‐/‐^ mice, and IRF7^‐/‐^ mice following KK2DP7 training and subsequent tumor or LPS inoculation for 48 h. In normal mice, KK2DP7 training resulted in a significant upregulation of TLR2, MyD88, p‐65, p‐p65, STAT1, IRF7, and p‐IRF7 expression, as well as an increase in IFN‐α/β/γ secretion levels compared to the untrained group (Figure 8e–h; Figure S8, Supporting Information). However, in TLR2^‐/‐^ mice, there were no significant changes in the expression of MyD88, p‐65, p‐p65, STAT1, IRF7, and p‐IRF7, or the secretion levels of IFN‐α/β/γ after KK2DP7 training (Figure 8f–i). Similarly, in IRF7^‐/‐^ mice, while TLR2 expression was upregulated after KK2DP7 training, there were no significant differences in the secretion levels of IFN‐α/β/γ compared to the untrained group (Figure 8g–j). These initial findings indicate that KK2DP7 activates TLR2, which in turn leads to the activation of IRF7 and the subsequent secretion of cytokines such as interferons. To provide stronger evidence supporting the role of KK2DP7 in activating TLR2 and IRF7 to promote antitumor immune responses through cytokine secretion, we evaluated the antitumor effects of KK2DP7 training in TLR2^‐/‐^ and IRF7^‐/‐^ mice. The results indicated a significant reduction in the antitumor effects of KK2DP7 training in both TLR2^‐/‐^ and IRF7^‐/‐^ mice (Figure 8k–n; Figure S6a–d, Supporting Information), thereby providing stronger evidence for the proposed mechanism.

In recent years, there has been a growing body of evidence in the literature demonstrating that the combined use of immunostimulatory agents and immune checkpoint inhibitor antibodies can achieve more significant therapeutic effects.^[^ ^5a,b^ ^]^ In light of these observations, we sought to ascertain the anti‐tumor effect of combining KK2DP7 with anti‐PD‐1 antibody. The experimental results demonstrated that the combined use of KK2DP7 and anti‐PD‐1 antibody exhibited superior anti‐tumor effects in the B16 model (as illustrated in Figure 8o–r; Figure S6d, Supporting Information). This discovery further substantiates the efficacy of combining immunostimulatory agents with immune checkpoint inhibitors in impeding tumor growth, offering a valuable reference for future clinical trials.

For safety evaluation, we performed an analysis to detect the presence of anti‐nuclear antibodies (ANA) and anti‐double‐stranded DNA (dsDNA) antibodies in mice after administration of the KK2DP7 training vaccine regimen. The results of this analysis showed no statistically significant difference in antibody levels compared to normal controls (Figure S9a,b, Supporting Information). Furthermore, histopathological examination of the tissue samples did not reveal any abnormal findings or pathological changes (Figure S9c, Supporting Information).

## 3 Discussion

Current immunotherapies predominantly target the adaptive immune system.^[^ ^17^ ^]^ However, emerging evidence suggests innate immune cells also exhibit anti‐tumor potential. Trained innate immunity‐induced through innate immune cell modulation‐mediates enhanced responsiveness to secondary challenges, warranting further investigation as an adjuvant tumor immunotherapy. Strategies aimed at reprogramming innate immune cells to adopt or reinforce an anti‐tumor phenotype have emerged as promising therapeutic avenues, either alone or combined with adaptive immunity‐targeted approaches.^[^ ^18^ ^]^ For example, Bacillus Calmette‐Guérin (BCG) treats bladder cancer via myeloid cell epigenetic reprogramming to enhance lymphocyte responses.^[^ ^8^, ^19^ ^]^ Similarly, β‐glucan induces anti‐tumor immunity by training neutrophils and synergizes with immune checkpoint inhibitors.^[^ ^5a^ ^]^ Another study demonstrated that β‐glucan triggers bone marrow pluripotent progenitor cell epigenetic reprogramming, amplifying anti‐tumor effects when combined with checkpoint inhibitors.^[^ ^5b^ ^]^ Collectively, these findings highlight how trained immunity agonists can empower innate immune cells to combat tumors. Despite growing recognition of trained immunity, the repertoire of known agonists remains limited, and their mechanisms are incompletely understood. Thus, developing novel agonists and elucidating their modes of action are critical priorities.

Adjuvant‐induced trained immunity constitutes a key component of adjuvant mechanisms. Here, we employed KK2DP7‐a dendrimer peptide derived from the immunomodulatory antimicrobial peptide DP7‐to establish protocols for studying adjuvant‐mediated enhancement of anti‐tumor immunity via trained innate immunity. Intravenous KK2DP7 administration conferred tumor growth resistance in mice. While literature reports describe subcutaneous, intraperitoneal, and intravenous routes for trained immunity agonists.^[^ ^5^, ^20^ ^]^ we selected intravenous administration based on preliminary biodistribution studies. Specifically, we compared splenic and bone marrow accumulation of Cy5‐labeled KK2DP7—key target organs for trained immunity—following subcutaneous, intramuscular, and intravenous delivery. Intravenous administration achieved optimal splenic accumulation (Figure S6, Supporting Information), aligning with reports that irregularly shaped particles > 200 nm preferentially localize to the spleen,^[^ ^21^ ^]^ whereas smaller nanoparticles (80–150 nm) access bone marrow through vascular endothelial gaps.^[^ ^22^ ^]^ KK2DP7 particles (≈360 nm) exhibited pronounced splenic accumulation, consistent with size‐dependent targeting trends. Subsequent analyses revealed elevated secretion of trained immunity‐associated cytokines (TNF‐α, IL‐1β, IL‐6, and IFN‐γ) in KK2DP7‐treated splenocytes upon LPS rechallenge. Our cytokine selection (TNF‐α, IL‐1β, IL‐6, IFN‐γ) was informed by prior literature,^[^ ^5^, ^23^ ^]^ with type I interferon added later based on sequencing data.

Based on the above observations, it is hypothesized that KK2DP7 may facilitate the training of splenic cells to mount a robust anti‐tumor immune response. To investigate this hypothesis, we surgically excised the spleens of mice. Notably, intravenous administration of KK2DP7 failed to effectively attenuate tumor growth in splenectomized mice, indicating that the target cells responsive to KK2DP7‐induced training reside within the splenic compartment. Furthermore, using lymphocyte isolation, flow cytometry, and magnetic bead sorting techniques, we confirmed that CD11b+ cells within the non‐lymphocyte fraction of the spleen, following KK2DP7 administration, are capable of secreting cytokines characteristic of trained immunity upon secondary stimulation with LPS. Additionally, when CD11b+ cells from the non‐lymphocyte layer of the spleen of mice trained with KK2DP7 were transferred to naïve mice, the recipient mice exhibited anti‐tumor growth capabilities. These findings collectively suggest that the primary target cells for immune anti‐tumor training mediated by KK2DP7 are CD11b+ cells residing in the non‐lymphoid layer of the mouse spleen.

To further elucidate the mechanistic basis of how KK2DP7 activates these CD11b+ cells for anti‐tumor activity, we conducted transcriptome and ATAC sequencing studies, along with validation experiments, on CD11b^+^ cells isolated after immune training with KK2DP7. For the selection of sequencing technology in our subsequent mechanistic studies, we opted for a combined approach of ATAC‐seq and transcriptomics, referencing relevant literature for guidance. Although much of the current literature suggests that trained immunity involves epigenetic and metabolic reprogramming, the majority of studies still favor RNA sequencing or a combination of RNA and ATAC‐seq to decipher the underlying mechanisms of trained immunity.^[^ ^3^, ^11^, ^24^ ^]^ Our approach aims to provide a more comprehensive understanding of the molecular processes involved in KK2DP7‐mediated immune training and its anti‐tumor effects. The sequencing and biological validation results demonstrated the activation of immune responses and cytokine secretion pathways in CD11b^+^ cells within the non‐lymphoid layer of the spleen following training with KK2DP7. Additionally, the results indicated a significant enhancement of CD11b^+^ cell proliferation, phagocytosis, and tumor cell killing abilities. Further investigation of signaling pathways revealed that phenotypic alterations in CD11b^+^ cells are regulated by the TLR2‐IRF7 signaling axis. Following immune training with KK2DP7 and tumor inoculation, TLR2 and IRF7 were significantly activated in CD11b^+^ cells within the non‐lymphocyte layer of the mouse spleen, accompanied by a significant increase in interferon secretion. In TLR2‐/‐ and IRF7‐/‐ mice, the anti‐tumor efficacy and interferon secretion induced by KK2DP7‐mediated training immunity were significantly reduced. Moreover, the combination of KK2DP7 and immune checkpoint inhibitors demonstrated superior anti‐tumor efficacy. These findings are consistent with those reported in other literature.^[^ ^5a,b^ ^]^

While prior studies established the role of β‐glucan or BCG in inducing trained immunity against infections,^[^ ^1^, ^25^ ^]^ our work uniquely identifies KK2DP7 as the first synthetic peptide adjuvant capable of specifically programming splenic CD11b^+^ myeloid cells to drive sustained anti‐tumor immunity. Notably, we uncovered a non‐monocytic origin of anti‐tumor trained immunity: in contrast to the predominant focus on bone marrow‐derived monocytes/macrophages, we demonstrate that splenic CD11b^+^ cells—including neutrophils and dendritic cell subsets—serve as the primary mediators of KK2DP7‐induced training, challenging current paradigms. These mechanistic distinctions from existing trained immunity models highlight KK2DP7's potential as a precision‐trained immunity inducer.

An intriguing aspect that warrants further exploration is the interaction between the activated innate immune system and the adaptive anti‐tumor immune response. Our results suggest that KK2DP7‐mediated activation of the splenic CD11b^+^ subpopulation triggers a cytokine secretion cascade that may critically bridge innate and adaptive immunity. These cytokines likely play a pivotal role in shaping the tumor microenvironment, promoting the activation and differentiation of adaptive immune cells, and ultimately enhancing anti‐tumor responses. The activation of innate immune cells by KK2DP7 significantly influences subsequent adaptive immunity, particularly the activation and proliferation of CD8^+^ T cells and B cells. Upon stimulation, innate immune cells secrete key cytokines such as TNF‐α and IFN‐γ, which are pivotal in shaping the adaptive immune landscape. This cytokine milieu fosters an environment conducive to robust anti‐tumor responses, amplifying adaptive immune efficacy. Furthermore, within the tumor microenvironment, the interplay between innate and adaptive immunity is vital, as innate immune cells modulate adaptive immune cell functions. Such interaction ensures the adaptive immune system is primed to efficiently recognize and eliminate tumor cells, underscoring its necessity for mounting effective anti‐tumor responses.

The potential applications of training immunity agonists in disease treatment are extensive and profound, particularly in the realm of immunotherapy targeting specific pathogens or tumors, where they have exhibited considerable promise. These agonists elicit and augment the immune system's response capabilities, thereby facilitating more effective disease resistance and potentially altering the disease trajectory. In the context of immunotherapy, training immunity agonists can be tailored to target specific pathogens or tumors by activating specific immune cells or signaling pathways, thereby enhancing disease resistance. For example, in the management of chronic infectious diseases, training immunity agonists can aid the immune system in better recognizing and eliminating pathogens, thus diminishing disease recurrence and progression.^[^ ^1^, ^2^ ^]^ In the realm of oncological therapy, these agonists can stimulate the immune system to target and attack tumor cells, augmenting antitumor immune responses and ultimately enhancing treatment outcomes. Additionally, training immunity agonists can serve as adjuvant therapy when combined with other treatment modalities. For instance, during chemotherapy or radiotherapy, the incorporation of training immunity agonists may mitigate side effects, improve patient tolerance, and further enhance treatment efficacy. This combined therapeutic approach capitalizes on the strengths of diverse treatment strategies to achieve superior overall therapeutic outcomes.

Nevertheless, despite the extensive potential applications of training immunity agonists, several challenges and limitations remain to be addressed. First, the selection of an appropriate dosage is critically important. A dosage that is too low may be insufficient to elicit the desired therapeutic effect, whereas a dosage that is too high may induce adverse reactions. Therefore, comprehensive dosage studies are essential for various diseases and patient populations to establish optimal dosing regimens. Second, the mode of administration must be carefully considered. The route of administration can influence the distribution, metabolism, and subsequently, the therapeutic efficacy of the agonists in the body. Consequently, determining the optimal mode of administration is crucial for maximizing the efficacy of training immunity agonists. Finally, the long‐term safety profile of training immunity agonists is a significant concern. Although these agonists can augment immune system function, prolonged and excessive stimulation may lead to immune dysregulation or the onset of autoimmune diseases.^[^ ^26^ ^]^ Therefore, it is imperative to rigorously monitor patients' immune status and implement appropriate preventive strategies when utilizing these agonists. In addition, it is critical to acknowledge the inherent disparities between mouse models and human applications. Although KK2DP7 demonstrates efficacy in murine tumor growth assays, physiological and immunological differences between species may complicate clinical translation. The dosage and route of administration optimized for mice may require adjustment in human trials to ensure safety and efficacy. Additionally, the long‐term effects and potential side effects of KK2DP7 remain underexplored in preclinical studies, necessitating caution when extrapolating results to patients. Patient heterogeneity—including age, sex, genetic background, and comorbidities—may also significantly influence therapeutic outcomes. Future research will prioritize clinical studies across diverse populations to rigorously assess KK2DP7's safety and efficacy. Addressing these limitations will clarify the translational potential of our findings and advance the application of trained immunity in anti‐tumor therapy.

In conclusion, training‐immunity agonists hold significant promise as therapeutic tools for disease treatment. However, significant challenges and limitations must be addressed. As research advances, future efforts will likely yield a broader repertoire of training‐immunity agonists, expanding their applicability across diverse diseases. Furthermore, trained immunity may evolve into a transformative strategy for improving existing vaccines or designing novel formulations that simultaneously induce classical adaptive immune memory and innate immune memory. This dual approach could prove invaluable during future pandemics, positioning trained‐immunity vaccines as a groundbreaking frontier for enhancing protection against emerging pathogens.

## 4 Experimental Section

### Cells and Animals

Roswell Park Memorial Institute (RPMI) 1640 Medium containing 100 units mL^−1^ streptomycin and penicillin (PS) and 10% fetal bovine serum (FBS, NEST Biotechnology) was used to culture splenic primary cells and EG7 cells (American Type Culture Collection, Manassas, VA, USA). Dulbecco's Modified Eagle Medium (DMEM) containing 100 units mL^−1^ streptomycin and penicillin (PS) and 10% fetal bovine serum (FBS) was used to culture CT26, LL2, 293T, and B16‐F10 cells (American Type Culture Collection, Manassas, VA, USA). All cells were cultured in a cell incubator containing 5% CO_2_ at 37 °C. RPMI 1640 medium, DMEM medium, and PS were all purchased from Thermo Fisher Scientific. Female six‐ to eight‐week‐old C57BL/6J mice were purchased from Beijing Vital River Laboratory Animal Technology Co., Ltd. TLR2^‐/‐^ mice were maintained by the State Key Laboratory of Biotherapy, Sichuan University. IRF7^‐/‐^ mice were purchased from the Model Animal Research Center. All animal procedures were approved and controlled by the Institutional Animal Care and Treatment Committee of Sichuan University and conducted according to the Animal Care and Use Guidelines of Sichuan University (Ethical number: 20240116004).

### Preparation of the KK2DP7

KK2DP7 ((VQWRIRVAVIRK)_2_K) was synthesized, purified (> 95%), and verified by Kotide Biotechnology Co., Ltd. (Shanghai, China). The HPLC and MS results were obtained from Kotide Biotechnology Co., Ltd. (Shanghai, China) (Figure S7c,d, Supporting Information). The morphological characteristics of KK2DP7 were examined by using TEM (H6009IV, Hitachi, Tokyo, Japan) (Figure S7a, Supporting Information). The diameter was measured by a Zetasizer Nano ZS (Malvern Panalytical Co. Ltd.). All results were the means from three experiments (Figure S7b, Supporting Information).

### Tumor Models

To assess the impact of dosage on the antitumor efficacy of KK2DP7‐induced trained immunity, mice (n = 6) were administered intravenous injections of KK2DP7 at concentrations of 0.25, 0.5, 1, and 2 mg kg^−1^ in 100 µL of PBS or PBS alone on days ‐6, ‐3, and 0. Subsequently, the mice were inoculated subcutaneously with 1 × 10^6^ EG7 tumor cells suspended in 100 µL of PBS. To evaluate the influence of administration frequency on the antitumor response elicited by KK2DP7‐induced trained immunity, mice (n = 6) were given intravenous injections of KK2DP7 at a dose of 1 mg kg^−1^ in 100 µL of PBS or PBS alone on day ‐6, or a combination of days ‐6, ‐3, or ‐6, ‐4, ‐2, along with subcutaneous inoculation of 1 × 10^6^ EG7 tumor cells in 100 µL of PBS on day 0. β‐Glucan served as a control and was administered subcutaneously to mice at a dose of 1 mg in 100 µL of PBS on day ‐7. To confirm the antitumor effects of KK2DP7‐induced trained immunity across multiple prophylactic tumor models, mice (n = 6) were treated with intravenous injections of KK2DP7 at 1 mg kg^−1^ in 100 µL of PBS or PBS alone on days ‐6, ‐4, ‐2, followed by subcutaneous inoculation with 1 × 10^6^ tumor cells (EG7, CT26, LL2) suspended in 100 µL of PBS on day 0. To validate the antitumor effects of KK2DP7‐induced trained immunity in therapeutic tumor models, mice (n = 6) were first inoculated subcutaneously with 1 × 10^6^ tumor cells (EG7, CT26, LL2) in 100 µL of PBS on day 0. Subsequently, the mice were treated with intravenous injections of KK2DP7 at 1 mg kg^−1^ in 100 µL of PBS or PBS alone on days 2, 4, and 6. Throughout all animal experiments described above, the tumor volume, body weight, and survival rate of the mice were closely monitored and recorded. Tumor volume was calculated using the formula: Tumor volume = 4/3 × π × (length/2) × (width/2) × (width/2). Mice were euthanized once their tumor volumes reached 1500 mm^3^ to ensure ethical treatment and alleviate any undue suffering.

To investigate whether KK2DP7 retains its antitumor activity following splenectomy in mice, the spleens of mice (*n* = 6) were surgically removed on day ‐20. Subsequently, these mice were administered intravenous injections of KK2DP7 (1 mg kg^−1^ in 100 µL PBS) or PBS on days ‐6, ‐4, ‐2. On day 0, the mice were inoculated subcutaneously with EG7 tumor cells (1 × 10^6^) suspended in 100 µL of PBS. To assess whether CD11b^+^ cells in the non‐lymphocytic layer of the spleen, trained by KK2DP7, contribute to tumor resistance in mice, a cohort of mice were treated with intravenous injections of KK2DP7 (1 mg kg^−1^ in 100 µL PBS) or PBS on days ‐8, ‐6, ‐4. On day ‐2, these mice were sacrificed to isolate CD11b^+^ cells from the non‐lymphocytic layer of the spleen. Separately, another group of mice was intravenously infused with the isolated CD11b^+^ cells (2 × 10^6^) in 100 µL of PBS on day ‐2. Subsequently, on day 0, both groups of mice were subcutaneously inoculated with EG7 tumor cells (1 × 10^6^) in 100 µL of PBS. Throughout all animal experiments described above, the tumor volume, body weight, and survival rate of the mice were closely monitored and recorded. Tumor volume was calculated using the formula: Tumor volume = 4/3 × π × (length/2) × (width/2) × (width/2). Mice were euthanized once their tumor volumes reached 1500 mm^3^ to ensure ethical treatment and alleviate any undue suffering.

To validate the mechanism by which KK2DP7 activates training immunity, on day 0, wild‐type, TLR2^‐/‐^, and IRF7^‐/‐^ mice (n = 6 per group) were inoculated subcutaneously with tumor cells (B16‐F10, 5 × 10^5^) suspended in 100 µL of PBS. Subsequently, these mice received intravenous injections of either KK2DP7 (1 mg kg^−1^ in 100 µL PBS) or PBS on days 2, 4, and 6. To assess the antitumor effects of training immunity combined with anti‐PD‐1 antibody, mice were inoculated subcutaneously with tumor cells (B16‐F10, 5 × 10^5^) in 100 µL of PBS. These mice were then treated with intravenous injections of KK2DP7 (1 mg kg^−1^ in 100 µL PBS) on days 2, 4, and 6. Additionally, intraperitoneal injections of 200 µg of anti‐PD‐1 antibody were administered on days 3, 6, 10, and 13. Throughout all of the aforementioned animal experiments, the tumor volume, body weight, and survival rate of the mice were carefully monitored and recorded. Tumor volume was calculated using the formula: Tumor volume = 4/3 × π × (length/2) × (width/2) × (width/2). Mice were euthanized once their tumor volumes reached 1500 mm^3^ to ensure ethical handling and minimize suffering.

### PBMC Extraction

Female C57BL/6J mice (n = 3) were bled into EDTA anticoagulant tubes for blood collection. Peripheral blood mononuclear cells (PBMCs) were then isolated using a mouse peripheral blood monocyte isolation kit (Solarbio, China). The isolated PBMCs were washed twice with 1×PBS (phosphate‐buffered saline) to prepare them for further use.

### Mouse Bone Marrow Primary Cell Extraction

Primary cells from the bone marrow of C57BL/6J female mice were obtained (n = 3). Briefly, the bone marrow cells were treated with red blood cell lysis buffer, followed by the addition of fresh RPMI 1640 complete medium to prepare them for further use.

### Extraction of Spleen Cells, Separation of Spleen Lymphocytes, Sorting of Spleen CD11b^+^ Cells

Spleen cells were isolated from C57BL/6J female mice (n = 3). Briefly, the spleen was placed on a 70 µm sieve and gently crushed using the plunger of a syringe to collect the primary spleen cells for subsequent experiments. For the separation of splenic lymphocytes, the spleen cells were suspended in mouse splenic lymphocyte separation solution (Beijing Solarbio Science and Technology Co., Ltd.) and separated according to the manufacturer's instructions. Cells from both the lymphocyte and non‐lymphocyte layers of the spleen were collected for further use. To isolate CD11b^+^ cells, the non‐lymphocyte layer cells were treated with red blood cell lysate (Solarbio, China) and incubated with CD11b^+^ magnetic beads (Thermo, USA). Subsequently, column‐based sorting was performed to separate CD11b^+^ and CD11b^−^ cells, which were then collected for subsequent experiments.

### Enzyme‐Linked Immunosorbent Assay (ELISA) Analysis

To ascertain the locus of KK2DP7‐induced training immunity, KK2DP7 (0, 1 mg kg^−1^) was administered three times, with a two‐day interval between each administration. Two days after the final administration of KK2DP7, spleen cells (5 × 10^5^) (n = 3), bone marrow cells (5 × 10^5^) (n = 3), and peripheral blood mononuclear cells (PBMCs) (5 × 10^5^) (n = 3) were isolated and cultured in a 24‐well plate. These cells were then stimulated with LPS (100 ng mL^−1^) for a duration of 24 h. Subsequently, the culture supernatants were harvested and assayed for the quantitation of TNF‐α, IL‐6, IL‐1β, and IFN‐γ utilizing ELISA kits (Elabscience, China).

To validate the characteristic response of trained immunity in vivo, KK2DP7 (0, 1 mg kg^−1^) was administered three times, with a two‐day interval between each administration. Two days after the last administration of KK2DP7, the lymphocyte layer cells of the spleen (5 × 10^5^), non‐lymphocyte layer cells of the spleen (5 × 10^5^), CD11b^+^ and CD11b^−^ cells from the non‐lymphocyte layer (1 × 10^5^) were collected and stimulated with LPS (100 ng mL^−1^), tumor cells (CT26, EG7, LL2, 1.25 × 10^5^) and normal cells (293T, 1.25 × 10^5^) or 40% culture supernatant of these cells (CT26, EG7, LL2, 293T) (n = 3). Subsequently, the culture supernatants were collected after 24 h and assayed for the content of TNF‐α, IL‐6, IL‐1β, and IFN‐γ using ELISA kits (Elabscience, China).

To validate the characteristic response elicited by in vitro training immunization, the lymphocyte layer cells of the spleen (5 × 10^5^), non‐lymphocyte layer cells of the spleen (5 × 10^5^), CD11b^+^ and CD11b^−^ cells from non‐lymphocyte layer (1 × 10^5^) were collected and stimulated with KK2DP7 (1 mg mL^−1^) three times, with a two‐day interval between each stimulation. Two days after the last addition of KK2DP7, LPS (100 ng mL^−1^) was added to the cells. Then the culture supernatants were collected after 24 h and assayed for the content of TNF‐α, IL‐6, IL‐1β, and IFN‐γ using ELISA kits (Elabscience, China) (n = 3).

To validate the pathway of training immunity, CD11b^+^ cells (1 × 10^5^) were collected from the non‐lymphoid cell layer of KK2DP7‐trained wild‐type, TLR2^‐/‐^, IRF7^‐/‐^ mice, and then stimulated with LPS (100 ng mL^−1^) for 24 h. Subsequently, the culture supernatants were collected and assayed for the content of IFN‐α, IFN‐β, and IFN‐γ using ELISA kits (Elabscience, China) (n  =  3).

To assess whether the administration of KK2DP7 would induce the production of autoimmune antibodies in mice, we conducted an experimental validation study. Specifically, fifty days after the completion of the third round of KK2DP7 treatment, sera were collected from the mice. These sera were then subjected to analysis for the detection of mouse anti‐nuclear antibodies (Shanghai Sepeson Biotechnology Co., Ltd.) and mouse anti‐double‐stranded DNA‐IgG antibodies (Shanghai Kocheng Biotechnology Co., Ltd.).

### Flow Cytometry Assay

To validate target cell populations for KK2DP7‐trained immunity, it isolated cells from the lymphoid layer and non‐lymphoid layer of the spleens of mice after KK2DP7 training. KK2DP7 (0, 1 mg kg^−1^) was administered three times, with an interval of two days between each administration. Two days after the last administration of KK2DP7, cells from the lymphocyte layer of the spleen were stained with NK1.1‐PAC, TNF‐α‐PE for further flow cytometry analysis. Additionally, cells from the non‐lymphocyte layer of the spleen were stained with CD45‐APC‐Cy7, CD11b‐FITC, F4/80‐APC, CD11c‐PE‐Cy7, Ly6G‐Percp, Ly6C‐Pacora, and TNF‐α‐PE for further flow cytometry analysis (n = 3).

To verify the expression of TLR2 in CD11b^+^ cells in the non‐lymphoid layer of the spleen in mice, it isolated cells from the non‐lymphoid layer of the spleens of mice after KK2DP7 training. As before, KK2DP7 (0, 1 mg kg^−1^) was administered three times, with an interval of two days between each administration. Two days after the last administration of KK2DP7, spleen cells (5 × 10^5^) from non‐lymphocyte layer were isolated and stained with CD45‐percp‐cy5.5, CD11b‐FITC, TLR2‐APC for further flow cytometry analysis (n = 3).

To verify the proliferation of CD11b^+^ cells in the non‐lymphocyte layer of the mouse spleen after KK2DP7 training, KK2DP7 (0, 1 mg kg^−1^) was administered three times, with an interval of two days between each administration. Two days after the last administration of KK2DP7, spleen cells (5 × 10^5^) from the non‐lymphocyte layer were isolated and cultured in a 24‐well plate, followed by LPS (100 ng mL^−1^), tumor cells (CT26, EG7, LL2, 1.25 × 10^5^) stimulation for 24 h. Subsequently, the cells were collected and stained with CD45‐percp‐cy5.5, CD11b‐FITC for further flow cytometry analysis (n = 3).

To verify the phagocytic ability of CD11b^+^ cells in the non‐lymphocyte layer of the mouse spleen after KK2DP7 training, KK2DP7 (0, 1 mg kg^−1^) was administered three times, with an interval of two days between each administration. Two days after the last administration of KK2DP7, the spleen cells (5 × 10^5^) from non‐lymphocyte layer were isolated and cultured in a 24‐well plate, followed by stimulation with tumor cells (CT26, EG7, LL2, 1.25 × 10^5^) for 24 h. These tumor cells had been pre‐transfected with Cy3‐labeled siRNA negative control using Lipo2000. Subsequently, the cells were collected and stained with CD45‐percp‐cy5.5, CD11b‐FITC for further flow cytometry analysis (n = 3).

In all of the above experiments, flow cytometry was performed on a FACSAria SOPR (BD, USA) flow cytometer, and the data were analyzed using FlowJo 10.8.1 software. All antibodies used for flow cytometry were purchased from BD Biosciences.

### Transcriptomics Sequencing

KK2DP7 (1mg kg^−1^) was administered three times, with an interval of two days between each administration. Two days after the last administration of KK2DP7, CD11b^+^ cells from the non‐lymphocyte layer of the spleen were isolated. Then the cells were lysed with Trizol and sent to Personalbio Biotechnology Co. for RNA extraction and subsequent transcriptomics sequencing (n = 3). The analysis and mapping of the results were also done through the company's cloud platform. Protein interaction network analysis and the visualization of transcription factor regulatory networks were done using Cytoscape software.

### Assay for Transposase Accessible Chromatin Sequencing (ATAC) Sequencing

KK2DP7 (1 mg kg^−1^) was administered three times, with an interval of two days between each administration. Two days after the last administration of KK2DP7, CD11b^+^ cells from the non‐lymphocyte layer of the spleen were isolated. Then the cells were cryopreserved and sent to Personalbio Biotechnology Co. for ATAC sequencing (n = 3). The analysis and mapping of the results were also done through the company's cloud platform. The visualization of transcription factor chromatin was completed using IGV software.

### Cell Killing Experiment

To verify the phagocytic ability of CD11b^+^ cells in the non‐lymphocyte layer of the mouse spleen after KK2DP7 training, KK2DP7 (0, 1 mg kg^−1^) was administered three times, with an interval of two days between each time. Two days after the last administration of KK2DP7, the CD11b^+^ cells (1 × 10^5^) from the non‐lymphocyte layer were isolated and cultured in a 24‐well plate, followed by tumor cells (CT26, EG7, LL2) at densities of 1 × 10^4^ and 2 × 10^4^ cells per well for 24 h. Subsequently, cell culture supernatants were collected and cytotoxicity was detected using a cytotoxicity LDH assay kit (MCE, US). Briefly, 50 uL of supernatant was added to 50 uL of assay working solution for a total of 15 min of incubation, followed by measurement of absorbance at 490 nm using an enzyme marker immediately after the addition of 50 µL of Stop Solution to each well. Various controls were set up as suggested in the kit instructions (n = 4).

### Western Blot Analysis

Total protein was extracted from CD11b^+^ cells isolated from the non‐lymphocyte layer of wild type, TLR2^‐/‐^, IRF7^‐/‐^ mice that were either trained or untrained with KK2DP7 (1 mg kg^−1^). The protein lysates (30 µg) were subjected to sodium dodecyl sulfate‐polyacrylamide gel electrophoresis and transferred onto membranes. Then, the membranes were probed with antibodies against GAPDH (SA30‐01), TLR2 (JM22‐41, HUABIO, China), IRF7 (SC0617, HUABIO, China), phospho‐IRF7 (Ser471/Ser472) (HUABIO, China), MyD88 (HUABIO, China), p65 (HUABIO, China), p‐p65 (HUABIO, China), STAT1 (HUABIO, China) and p‐STAT1 (HUABIO, China). After probing, the membranes were incubated with horseradish peroxidase (HRP)‐conjugated secondary antibody (Abcam, USA). Finally, a chemiluminescence system (Millipore, Massachusetts, USA) was used to visualize and photograph the target protein bands.

### Histological Analysis

After 50 days of immunization with the third round of treatment, the main organs were harvested and fixed immediately using 4% paraformaldehyde for 72 h. Then, the tissues were cut into thin slices of no more than 5 mm and placed in the embedding cassette, which was rinsed overnight using tap water. The subsequent tissue dehydration and embedding process was as follows: 1) The embedding cassettes with the tissue block were transferred to 75% ethanol and soaked overnight. 2) It was transferred to 85% ethanol and soaked for 30 min. 3) It was transferred to 95% ethanol and soaked twice for 15 min each time. 4) It was transferred to 100% ethanol and soaked three times for 15 min each time. 5) They were transferred to xylene solution and soaked twice for 15 min. 6) They were transferred to fresh paraffin solution three times for 30, 20, and 10 min. 7) They were transferred to fresh paraffin for embedding. Finally, the embedded tissue sections were dewaxed and rehydrated before being stained with Mayer's hematoxylin and eosin (H&E) according to the vendor's instructions (Solarbio, China).

### Statistical Analysis

All the values in the present study are presented as the mean ± S.D. Unless otherwise indicated in the Figure captions. One‐way analysis of variance was used for multiple comparisons (ANOVA) when more than two groups were compared, followed by post hoc Tukey's multiple comparison test, and Student's t‐test was used for two‐group comparisons. All the statistical analyses were carried out with the GraphPad Prism software package (PRISM 9.0; GraphPad Prism Software). Survival curves were obtained using the Kaplan–Meier method and compared by the log‐rank test. Statistical significance was set at a threshold of *P* < 0.05. Significant differences are denoted as follows: ^*^ *P* < 0.05, ^**^ *P* < 0.01, ^***^ *P* < 0.001, and ^****^ *P* < 0.0001.

## Conflict of Interest

The authors declare no conflict of interest.

## Author Contributions

Y.L. and Z.R. designed the study, and Z.R. was responsible for all experiments and articles; T.L., W.Y.S., Z.X.J., and D.Z.Y. helped Z.R. to perform the tumor model and the in vitro experiment. All authors read and approved the final manuscript.

## Supporting information



Supporting Information

## Data Availability

The data that support the findings of this study are available on request from the corresponding author. The data are not publicly available due to privacy or ethical restrictions.
