# Supplementary material for: KK2DP7 Stimulates CD11b+ Cell Populations in the Spleen to Elicit Trained Immunity for Anti‐Tumor Therapy
Source: Adv Sci (Weinh). 2025 Mar 27;12(23):2500032. doi: 10.1002/advs.202500032 (PMC12199376; doi:10.1002/advs.202500032)
Supplement: Supplementary file 1 — Supporting Information [file ADVS-12-2500032-s001.docx]

**Supplementary material**


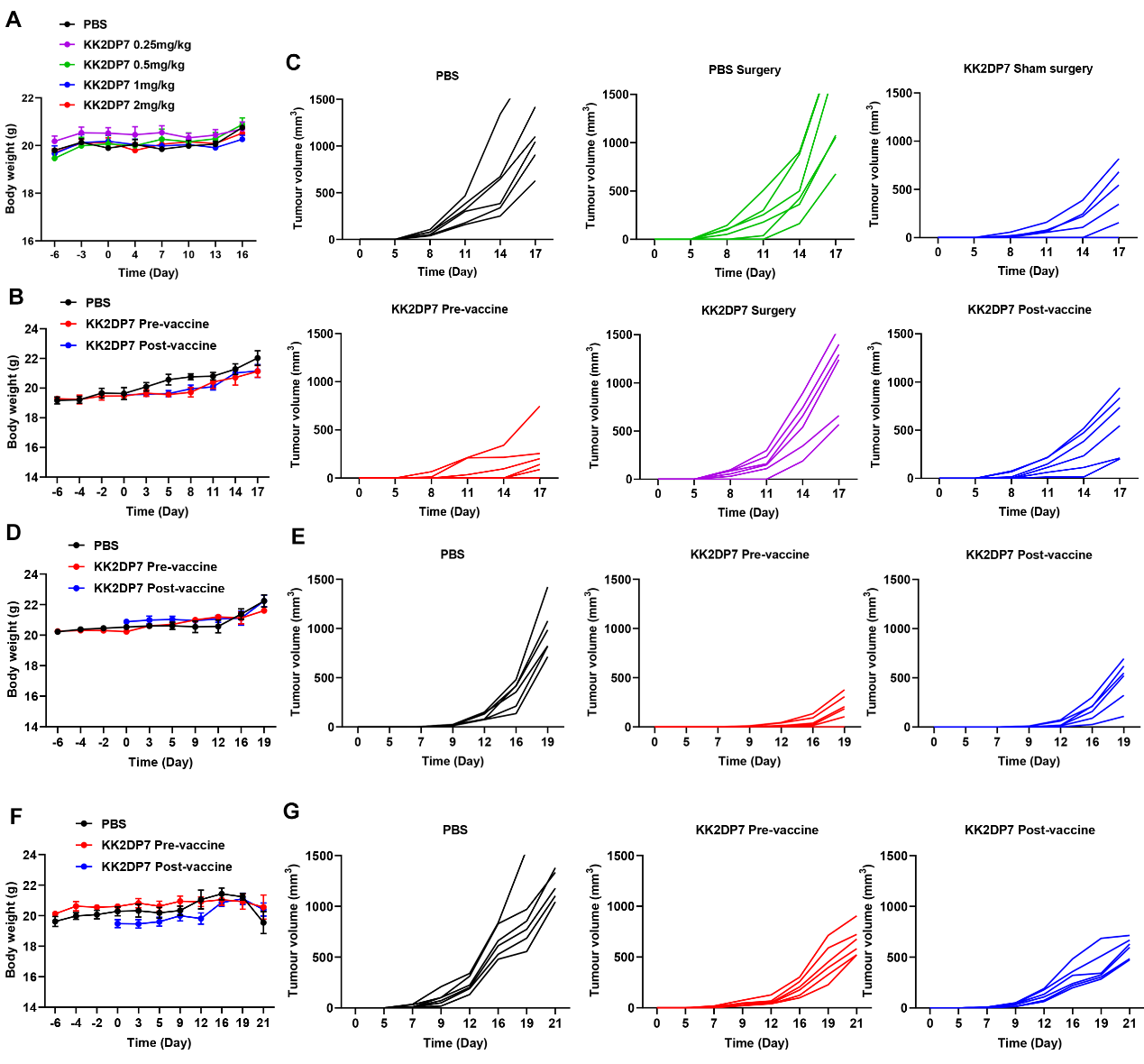


**Figure S1.** The administration of KK2DP7 has been shown to facilitate the resistance of mice to the growth of multiple tumors. a) Body weight of mice in different groups with different administration doses of KK2DP7. b) Body weight of mice of KK2DP7 pre- and post-administration in the EG7 tumor model. c) Individual tumor growth curves from Figure 1k and Figure 2e-2h. d) Body weight of mice of KK2DP7 pre- and post-administration in the CT26 tumor model. e) Individual tumor growth curves from Figure 1l. f) Body weight of mice of KK2DP7 pre- and post-administration in the LL2 tumor model. g) Individual tumor growth curves from Figure 1m.


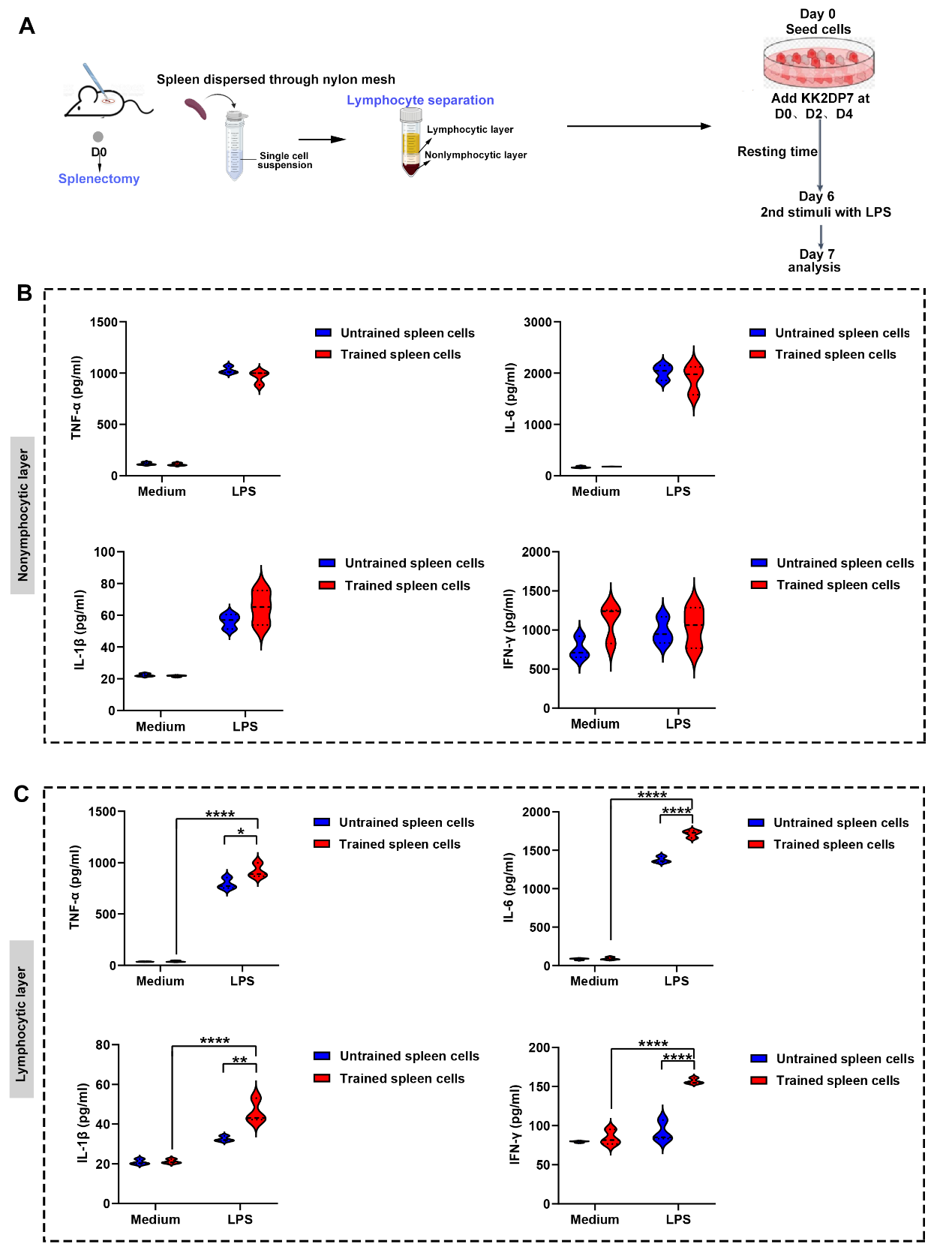


**Figure S2.** The target cells for training immunity induced by KK2DP7 are cells in the non-lymphocyte layer of the spleen. a) Schematic diagram of the verification of the site for KK2DP7 induced training immune response generation. b) Cells in the splenic lymphocyte layer of KK2DP7-trained and LPS-stimulated show no significant changes in the secretion of training-immunity-associated TNF-α, IL-1β, IL-6, and IFN-γ. c) Cells in the splenic non-lymphocyte layer of KK2DP7-trained and LPS-stimulated mice exhibited a significant upregulation of training-immunity-associated secretion of TNF-α, IL-1β, IL-6, and IFN-γ. All values presented in this figure are expressed as the mean ± s.d., unless otherwise indicated in the figure captions. Statistical analysis was performed using one-way ANOVA with Dunnett’s multiple comparison test. *P< 0.05, **P< 0.01, ****P< 0.0001.


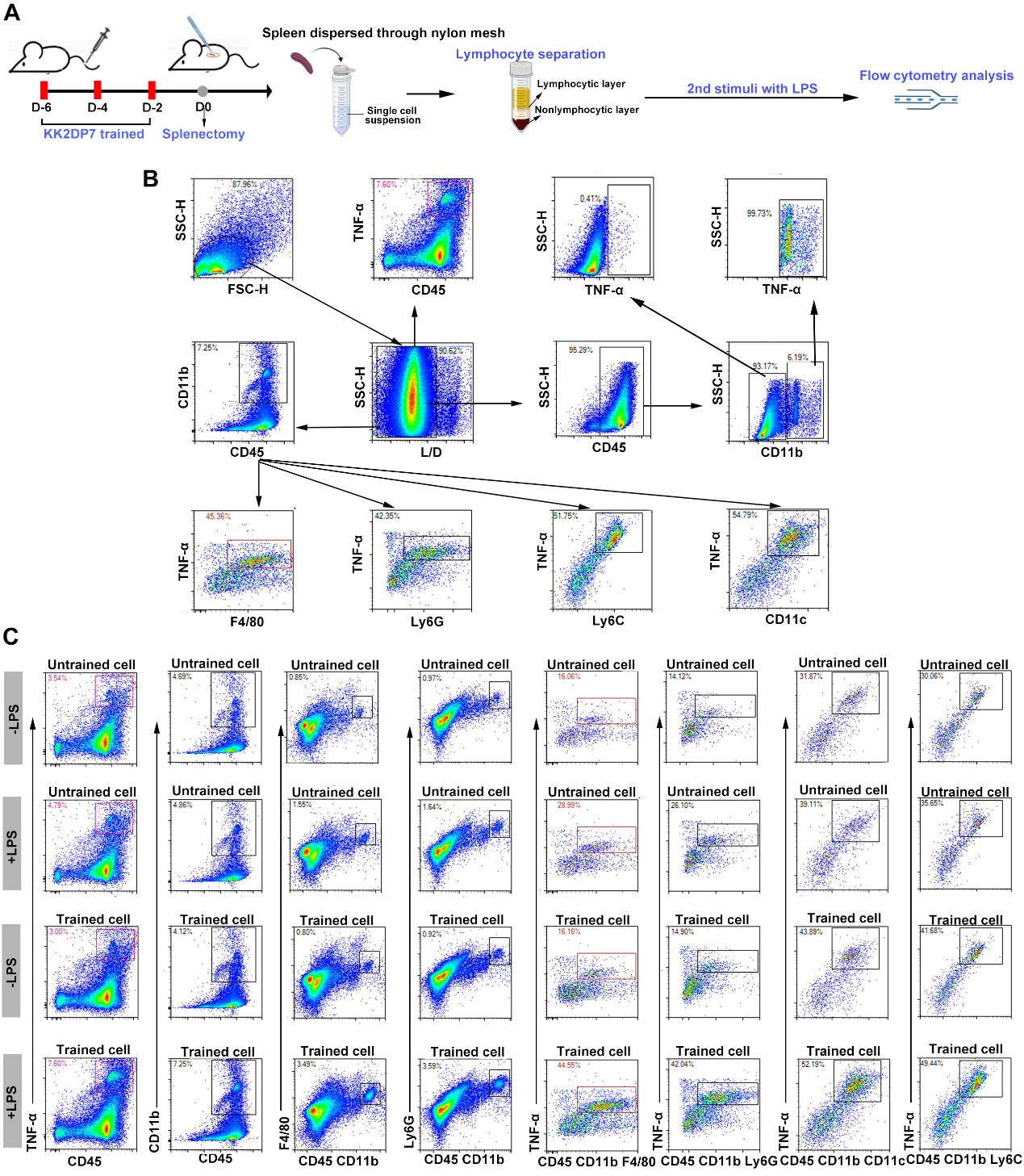


**Figure S3.** The target cells for training immunity induced by KK2DP7 are cells in the non-lymphocyte layer of the spleen. a) Validation flowchart of KK2DP7-induced training immunity in spleen target cell population. b) Flow cytometry gating strategy for the analysis of non-lymphocyte layer of the spleen. c) Representative flow diagram of training immunity induced by KK2DP7 through activation of CD11b^+^ cells in the splenic non-lymphoid cell layer.


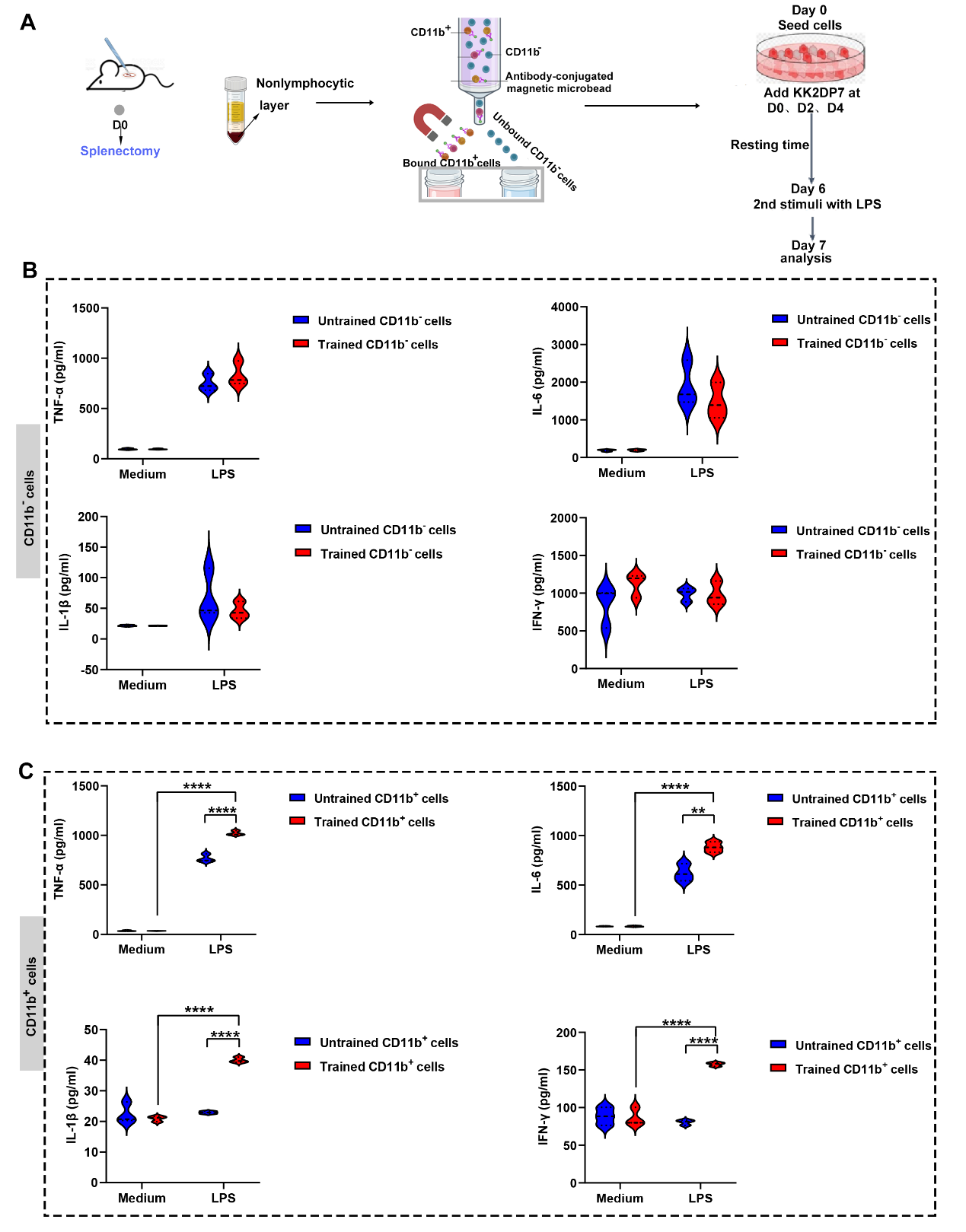


**Figure S4.** KK2DP7 induces the training of immunotherapy by activating CD11b^+^ cells in the non-lymphocytic layer of the spleen a) The validation flowchart of KK2DP7-induced training immunity in the spleen target cell population is presented below. b) CD11b^-^ cells in the splenic nonlymphocytic layer of KK2DP7-trained and LPS-stimulated animals did not exhibit any significant changes in the secretion of training-immunity-associated TNF-α, IL-1β, IL-6 and IFN-γ. c) CD11b^+^ cells in the splenic non-lymphocytic layer of KK2DP7-trained and LPS-stimulated animals exhibited a significant upregulation of TNF-α, IL-1β, IL-6, and IFN-γ secretion, which is indicative of training immunity. All values presented in this figure are expressed as the mean ± s.d., unless otherwise indicated in the figure captions. Statistical analysis was performed using one-way ANOVA with Dunnett’s multiple comparison test. **P< 0.01, ****P< 0.0001.


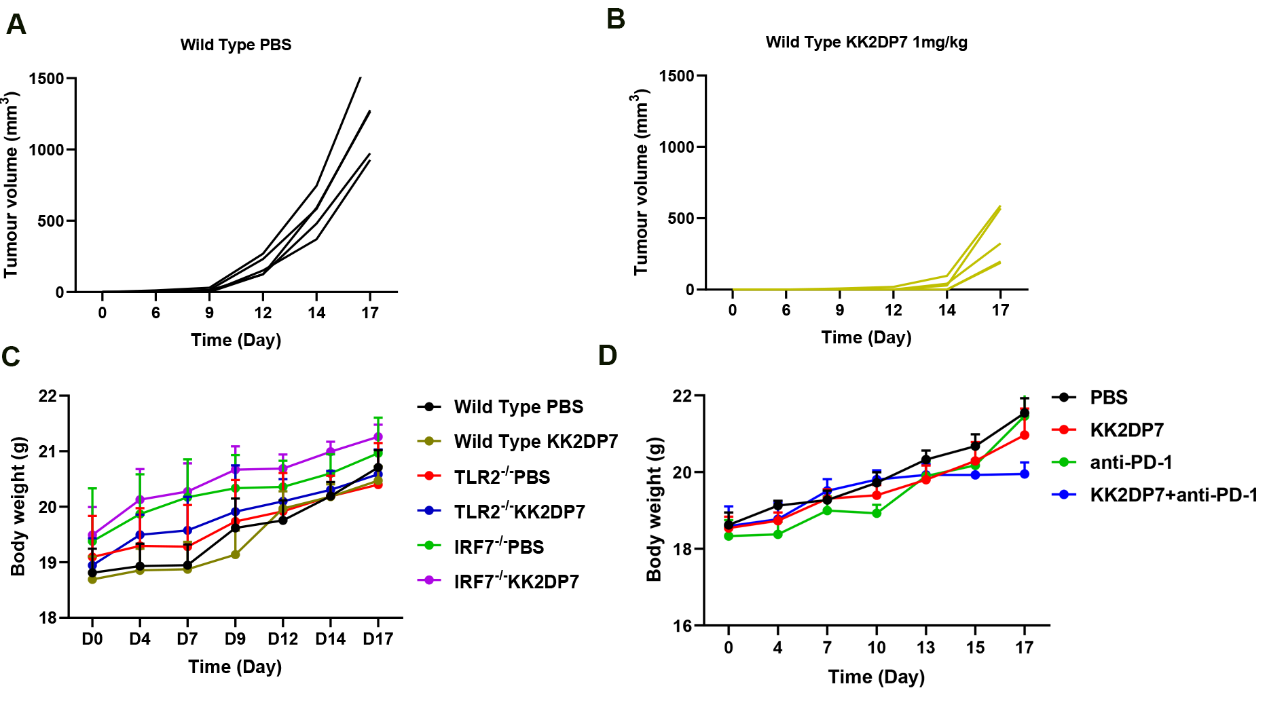


**Figure S5.** The induction of training immunity by KK2DP7 is dependent on the involvement of TLR2-IRF7 signaling. a-b) Individual tumor growth curves from Figure 8l. c) Body weight of mice from Figure 8l. d) Body weight of mice from Figure 8p.


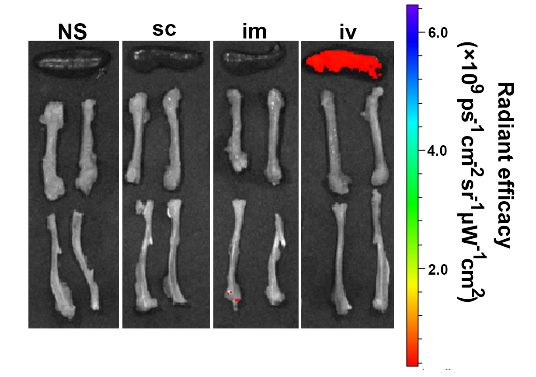


**Supplementary Figure 6.** In vivo spectrum imaging system (IVIS) fluorescence imaging of isolated spleens and bone marrows from mice at 24h after 1mg/kg of cy5-KK2DP7 subcutaneously (sc), intramuscularly (im), or intravenously (iv) injection.


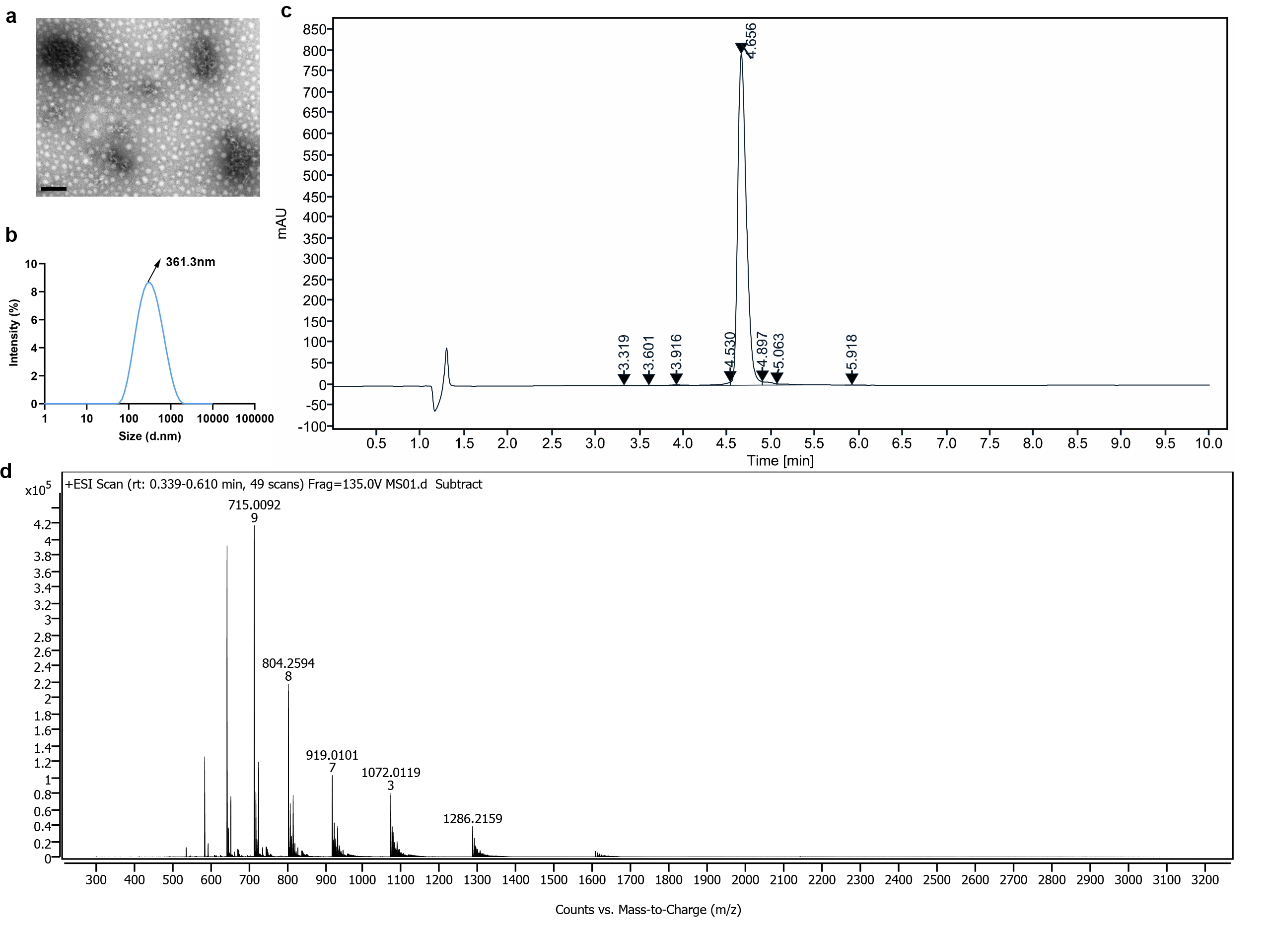


**Figure S7. HPLC, MS and particle size analysis of KK2DP7.** a) Morphology of KK2DP7 under transmission electron microscopy. b) Diameter of KK2DP7 (n = 3). c) High Performance Liquid Chromatography of KK2DP7. d) Mass Spectrometry (MS) of KK2DP7.


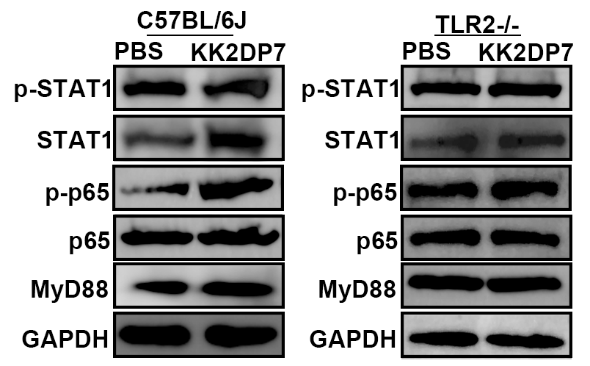


**Figure S8.** The validation of MyD88, p65, p-p65, STAT1 and p-STAT1 expression before and after immune training with KK2DP7 in wild-type mice, TLR2 knockout mice.


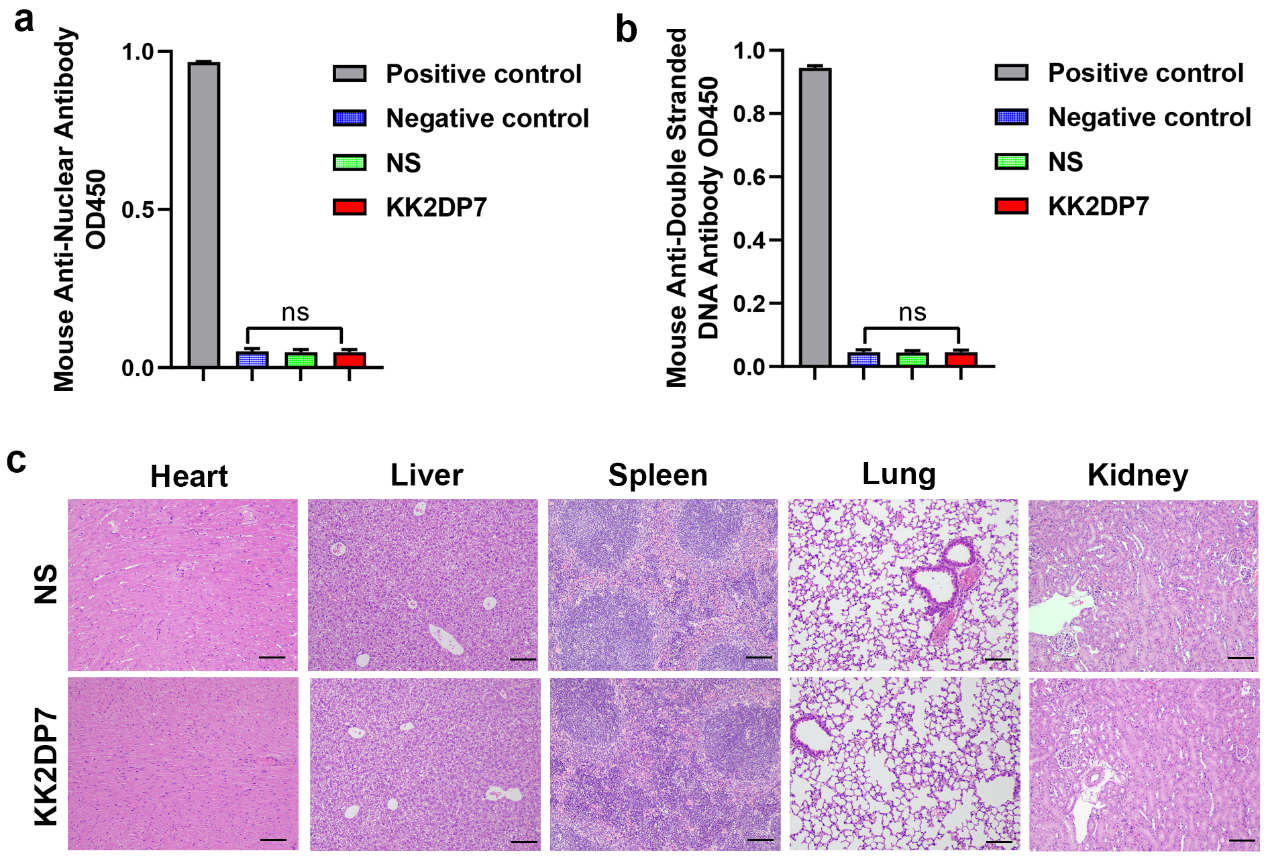


**Figure S9.** **Safety Evaluation.** a) Mouse serum anti-nuclear antibody assay. b) Mouse serum anti-double-stranded DNA antibody test. c) Results of HE staining of major organs (Scale bar, 100μm).
